# Supplementary material for: Polyphenol-stabilized coacervates for enzyme-triggered drug delivery
Source: Nat Commun. 2024 Aug 24;15:7295. doi: 10.1038/s41467-024-51218-8 (PMC11344779; doi:10.1038/s41467-024-51218-8)
Supplement: Supplementary file 1 — Supplementary Information [file 41467_2024_51218_MOESM1_ESM.pdf]

Supplementary Information for

Polyphenols-Stabilized Coacervates for Enzyme-Triggered Drug Delivery

Wonjun Yim[1], Zhicheng Jin[2], Yu-Ci Chang[1], Carlos Brambila[2], Matthew N. Creyer[2], Chuxuan Ling[2], Tengyu He[1], Yi Li[2], Maurice Retout[2], William F. Penny[3], Jiajing Zhou[2], Jesse V. Jokerst[1,2,4]

[1] Materials Science and Engineering Program, University of California San Diego, La Jolla, CA, 92093, USA

[2] Aiiso Yufeng Li Family Department of Chemical and NanoEngineering, University of California San Diego, La Jolla, CA, 92093, USA

[3] Division of Cardiology, VA San Diego Healthcare System, University of California San Diego, La Jolla, CA, 92093, USA

[4] Department of Radiology, University of California San Diego, La Jolla, CA, 92093, USA

*Correspondence and requests for materials should be addressed to
J.V.J. (jjokerst@ucsd.edu)

Table of Contents:

1. Supplementary Materials
2. Supplementary Methods
   2.1. Peptide synthesis
   2.2. Sulfo-Cy5.5-NHS conjugation
   2.3. Fluorogenic peptide synthesis
   2.4. TA-coumarin conjugates
   2.5. Encapsulation efficiency
   2.6. Limit of detection
   2.7. Colloidal stability experiment
   2.8. Enzyme kinetics for fluorogenic substrate
   2.9. Specificity test
   2.10. Heparin release confirmation
   2.11. Stability test in different biofluids and media
   2.12. ELISA test of F1+2 peptide
   2.13. Turbidity calculations
   2.14. Cytotoxicity test and cell staining of HEK 293
   2.15. General characterizations
3. Supplementary Notes: Thrombin
4. Supplementary Figures 1–38
5. Supplementary References

1. Supplementary Materials

Heparin sodium (from the porcine mucosa) was purchased from Henry Schein (NDC 63739-931-28). Human alpha-thrombin was purchased from Prolytix (HCT-0020) and stored at –20 °C for future use. Fresh human serum and plasma were obtained from Innovative Research and stored at –20 °C for future use. Cyanine3 maleimide (non-sulfonated, #21080), Sulfo-Cyanine5.5 NHS ester (#27320), and Cyanine5.5 NHS ester (#27020) were purchased from Lumiprobe (Cockeysville, MD). Pyridine (ACS reagent), acetic anhydride (ACS reagent), thioanisole (>99%), 2,2′-(ethylenedioxy)diethanethiol (EDDET, 95%), N,N-diisopropylethylamine (DIPEA, >99%), and triisopropylsilane (TIPS, >98%) were purchased from Tokyo Chemical Industry Co., Ltd (TCI). Bicine (>99%), Trizma® base (>99.9%), Trizma® hydrochloride (>90%), TFA (ReagentPlus®, 99% for cocktail solution), trifluoroacetic acid (TFA, HPLC grade, >99%), piperidine (ReagentPlus®, 99%), hemoglobin human (lyophilized powder), albumin from bovine serum (BSA), tannic acid (ACS reagent), citric acid (ACS reagent, (≥99%)), Triton™ X-100 (BioXtra), and automation compatible syringe filters (hydrophilic PTFE, 0.45 µm) were purchased from Sigma-Aldrich (St Louis MO). PEG-methoxy (M_w: 2000) was purchased from Laysan Bio (Arab, AL). Human umbilical vein endothelial cell (HUVEC) and human embryonic kidney (HEK) 293 cell lines were purchased from the American Type Culture Collection (ATCC, USA). ROS Detection cell-based assay kit (DCF-DA) was purchased from Cayman Chemical. Thrombin chromogenic substrate was purchased from Innovative Research (USA). Heparin fluorescein (Mw: 27k) was purchased from Creative PEGWorks (HP-201, USA). PrestoBlue cell viability reagent (A13262) was purchased from Thermo Fisher Scientific. Glucose, glutamine, and fibrinogen were purchased from Sigma-Aldrich (St Louis MO). Sodium hydroxide (NaOH, certified ACS), hydrochloric acid (certified ACS), sodium chloride (NaCl, certified ACS), urea (certified ACS), and hydrochloric acid (certified ACS) were purchased from Fisher Chemical (Waltham, MA). The purified recombinant SARS-CoV-2 main protease (M^{pro}) was provided by Dr. Hilgenfeld, University of Lübeck (Germany) and stored at –80 ºC in 20 mM Tris-HCl, pH 8.0, 150 mM NaCl, 1mM DTT, and 5% glycerol. N,N–dimethylformamide (DMF), acetonitrile (ACN, HPLC grade), ethyl ether (ACS certified), methylene chloride (DCM, certified ACS), dimethyl sulfoxide (DMSO, certified ACS), and uranyl acetate solution 2% were purchased from Fisher Scientific (Hampton, NH). Fmoc-Rink amide MBHA resin (0.55 mmol/g), Fmoc-protected amino acids, and hexafluorophosphate benzotriazole tetramethyl uranium (HBTU) were purchased from AAPPTec, LLC (Louisville, KY). Fmoc-L-propargylglycine (for click chemistry, #OR-2360) was purchased from Combi-blocks (San Diego, CA). 1% uranyl acetate solvent was purchased from Fisher Scientific (#224001, USA). Disposable reaction vessels and pressure caps for peptide-resin cleavage were obtained from Torviq Inc (Tucson, AZ). The SealPlate® film (MKCS0276) was purchased from EXCEL Scientific. Human Prothrombin Fragment 1+2 (F1+2) ELISA kit was obtained from abbexa (Cat. No: abx252419, UK). All reagents were used without further purification. Deionized water (18.2 MΩ·cm) purified with a Milli-Q Academic water purification system (IQ 7000) to make aqueous solutions. Glassware and stir bars were cleaned with aqua regia and washed with DI water at least three times before use.

2. Supplementary Methods

2.1. Peptide synthesis

Peptides were synthesized using the Fmoc-SPPS (solid-phase peptide synthesis) on Rink-amide-MBHA-resin (0.55 mmol/g) using an automated Eclipse™ peptide synthesizer (AAPPTec, Louisville, KY). Amino acids were linked to the resin under nitrogen conditions with 0.2 M Fmoc-amino acid, 0.2 M HBTU, 0.4 M DIPEA, and 20% (v/v) piperidine in DMF for each peptide synthesis cycle. Fmoc groups on the amino acids were removed using piperidine. HBTU and DIPEA were utilized for amide coupling. The resulting peptides were transferred to a syringe filter and washed with DCM to remove DMF. Chemical reactions such as click and acetylation on the peptide were performed on the resin before peptide cleavage. The crude peptides were then cleaved from the resin using a cocktail solution (5 mL) containing 5 mL of TFA (83%), 300 µL of H_2O (5%), 300 µL of thioanisole (5%), 300 mg of phenol (5%), and 150 µL of EDDET (2%). After 3h incubation, the resin was filtered, and the crude peptides were precipitated using a cold ethyl ether by three-time centrifugations (7,500 rpm, 5 min). The precipitated pellets were dried and re-suspended using 10 mL of ACN/H_2O mixtures (20–40% of ACN depending on the solubility of the pellets) for high-performance liquid chromatography (HPLC) purification. *Note*: Over 50% ACN can interrupt peptide purification because the absorbance peaks of ACN and peptides can be overlapped.

The crude peptides were purified using a Shimadzu LC-40 HPLC system equipped with an LC-40D solvent delivery module, a photodiode detector SPD-M40, and a degassing unit DGU-403. For each cycle, 2 mL of the crude peptides dissolved in ACN/H_2O mixtures was injected, and the C4 column (pore size: 5 µm, 20×200 mm, from SHMADZU) was used. Sample injection was monitored at 220 nm to collect the target peptide, and the wavelength at 680 nm was used for monitoring to collect sulfo-Cy5.5 conjugated peptides. The desired peptide was confirmed using an electrospray ionization mass spectrometry (ESI-MS) or/and matrix-assisted laser desorption/ionization (MALDI-TOF MS) from the first cycle. The purified target peptide was frozen at –80 ºC for lyophilization. All purified peptides achieved a minimum 90% purity, confirmed by ESI-MS or/and MALDI-TOF. The concentration of each peptide was calibrated using a NanoDrop™ UV-vis-spectrometer (Thermo Fisher Scientific, Waltham, MA). The 31-method in Nanodrop™ was used to calibrate the peptide concentration using an absorption of coefficient of ε_{205} (about 75 $mL \cdot mg^{-1} \cdot cm^{-1}$). MQ water was used as a blank. After concentration measurement, the purified peptides were dried using a vacufuge (Eppendorf, Germany) and stored at –20 ºC for future use.

2.2. Sulfo-Cy5.5-labeled C5 peptide (i.e., C7)

Briefly, C5 peptide (2 μmol) was mixed with sulfo-Cy5.5-NHS (1 μmol) in DMSO with 1% v/v triethylamine. The reaction was stirred for 3 h and covered with an aluminum foil to protect the dye against UV light. After a 3 h reaction, a vacufuge (Eppendorf, Germany) was used to dry DMSO at 60 °C. The dried pellet was re-dispersed using ACN/H_2O solvent for HPLC purification. The conjugation yield was ~40% after HPLC purification, and MALDI-TOF was used to confirm the mass peak shown in Supplementary Figure 13. *Note*: Sulfo-Cy5.5. without C5 conjugation cannot be encapsulated in the nano-coacervates due to its negatively charged nature.

2.3. Fluorogenic peptide synthesis (i.e., C8)

Cy5.5-peptide-Cy3. Briefly, an aqueous solution of the peptide (2 μmol, NH_2-YRLVPRGSYRC-Am, in DMSO) was mixed with TCEP (0.16 μmol) under gentle stirring for 30 min. After 30 min, Cy3-maleimide (2 μmol) was added to the above mixture for 3 h sealed with an aluminum foil. *Note*: The addition of a few drops of DI water may homogenize the reaction. After a 3 h reaction, the solvent was dried using a vacufuge, and the pellets were re-dispersed with ACN/H_2O for HPLC purification. MALDI-TOF was used to confirm the mass peak shown in Supplementary Figure 14.

Next, a solution of YRLVPRGSYRC-Cy3 (0.5 μmol in 950 μL of DMSO) was mixed with triethylamine (TEA, 10 μL, 1% v/v) and Cy5.5-NHS (1 μmol, in 50 μL of DMSO) under gentle stirring for 3 h sealed with aluminum foil for dye protections against the light. After a 3 h reaction, the sample was dried using a vacufuge (Eppendorf, Germany) at 60 °C. Then, the sample was re-dispersed using ACN and purified *via* HPLC. MALDI-TOF MS data was used to confirm the mass peak of the final product shown in Supplementary Figure 14.

All the above peptide-dye conjugates used the same HPLC protocol. The solvent gradient was carefully adjusted for hydrophobic dye purification. The peptide-dye conjugates were dissolved in ACN first and diluted with H_2O, depending on the conjugate's solubility. Then, 1 mL of the sample was injected into the C4 column and eluted at a flow rate of 3.0 mL/min over 60 min linear gradient from 10% to 90% ACN in water (with 0.05% TFA, HPLC grade). The sample was monitored at 220 nm (peptide), 550 nm (for Cy3), and 650 nm (for Cy5.5). The dye conjugates were quantified and aliquoted using an extinction value. Their molar extinction coefficients are $\varepsilon_{Cy3} = 1.5 \times 10^5\ M^{-1}cm^{-1}$ at $Abs_{555\ nm}$, and $\varepsilon_{Cy5.5} = 1.98 \times 10^5\ M^{-1}cm^{-1}$ at $Abs_{685\ nm}$.[1]

2.4. Synthesis of TA-coumarin conjugates and confocal imaging

Both TA and coumarin boronic acid were dissolved in DMSO and mixed at a 1:1 ratio with 0.5% TEA for 18 h at room temperature. After conjugation, the sample was dried using a vacufuge (Eppendorf, USA) at 60 °C. and re-dispersed in H_2O/ACN for HPLC purification. After purifying the conjugates, TA-coumarin was dried again using a vacufuge for future use.

For confocal imaging, 300 µL of heparin (100 U/ml) was first mixed with 300 µL of C5 peptide (1.3 mg/ml), forming micro-sized coacervates. After a 1 h of gentle shaking, 25 µL of TA-coumarin-TA conjugates (1.1 mg/ml) was added and incubated in the shaker overnight. The sample was then centrifuged at 3 x g for purification and re-dispersed in a 96-well plate for the confocal imaging.

2.5. Encapsulation efficiency

Briefly, the encapsulation efficiency was calibrated using a supernatant collected after the C5–heparin coacervation. After mixing heparin and peptides to form complex coacervates, the sample was centrifuged at 3 x g for 15 min to collect the supernatant. The supernatant which includes unreacted heparin was incubated with 100 µM of MB dyes to measure the changes in absorbance at 680 nm. Notably, the absorbance of MB dyes decreases as a function of heparin concentration from 0 to 10 U/ml because of MB–heparin aggregation (Supplementary Figure 9). Encapsulation efficiency (EE%) was calculated based on the following equation[2]:

$$EE(\%) = \frac{(Total\ heparin\ conc. - free\ heparin\ conc)}{Total\ heparin\ conc.}\ X\ 100$$

20 µL of MB dye (100 µM) was mixed with 60 µL of different heparin concentrations to measure the decreased absorbance at 666 nm (Supplementary Figure 9). At least three replicates were performed independently to collect the mean value and standard deviations. Likewise, 60 µL of the supernatant collected from the coacervate samples was mixed with 20 µL of MB dye (100 µM). The decreased absorbance was used to calibrate the encapsulation efficiency.

2.6. Limit of detection calculation

The limit of detection (LoD) was calibrated using the limit of blank (LoB).[3] The LoB indicates the highest signal generated from a sample that contains no analyte. LoB could be calculated using the mean ($mean_{blank}$) and standard deviation (SD_{blank}) of a blank sample with the following equation[3]:

$$LoB = mean_{\text{blank}} + 1.645\ (SD_{\text{blank}})$$

The LoD defines the lowest analyte concentration that could differ from the LoB. LoD is calculated based on the LoB and standard deviation of the lowest concentration samples ($SD_{\text{low concentration sample}}$) with the following equation[3]:

$$LoD = LoB + 1.645\ (SD_{\text{low concentration sample}})$$

Here, we used YRLVPRGSYR peptides (i.e., C5) with unfractionated heparin to form complex coacervates. Different concentrations of thrombin were incubated with nano-coacervates to measure the LoD. The buffer condition was Tris-HCl pH 8.5 with NaCl 150 mM at 37 ºC. The assay was then transferred to the 96-well plate, and the absorbance at 500 nm was measured for turbidity calibration. At least three independent replicates were performed to measure the mean value and standard deviation.

2.7. Colloidal stability in different pH and conditions

Briefly, $(YR)_2$–heparin coacervates of 1 mM were incubated at different pH values (2–14) for 1 h. We then measured the absorbance from 300 to 900 nm. 1 mM indicates the final peptide concentration used for coacervation. The turbidity was calibrated using the absorbance at 500 nm. Different pH was controlled using HCl and NaOH and measured using a pH meter.

The colloidal stability of nano-coacervates in different conditions including PEG2000, citric acid, urea, Triton-X, SDS, DMF, and DMSO. These were used to examine which interactions govern the stability of $(YR)_2$–heparin assemblies. Nano-coacervates were incubated in 10 µM of PEG2000, urea, Triton-X, citric acid (pH 2), and 70% of DMF and DMSO, respectively. The change in absorbance at 500 nm was measured before and after the incubation. At least three replicates of each experiment were performed to measure the average and standard deviation. Disassembly value was measured with the following equation[4]:

$$Disassembly(\%) = \frac{Turbidity_{before} - Turbidity_{after}}{Turbidity_{before}} \times 100$$

2.8. Enzyme kinetics for fluorogenic substrate

The kinetic model of an enzyme was developed by Michaelis[5] and Menten and it was further refined by Briggs-Hadane[6] with significant improvements. In brief, the enzyme (*E*) and substrate (*S*) reversibly form a reversible enzyme-substrate complex (*ES*), followed by the dissociation of the intermediate complex to produce the product along with free enzyme, which can be expressed as follows:

$$E + S \xrightarrow{k_{on}} ES \xrightarrow{k_{cat}} E + P$$

$$E + S \underset{k_{off}}{\longleftarrow} ES \underset{k_{-cat}}{\longleftarrow} E + P$$

where k_{on}, k_{off}, k_{cat}, and k_{-cat} are rate constant. The relationship between the initial velocity of the complex formation (v) and the substrate concentration ($[S]_0$) is known at the classical Michaelis-Menten (MM) equation[7]:

$$= \frac{v_{max} \cdot [S]_0}{K_M + [S]_0}$$

where v_{max} indicates the maximum velocity and K_M is the MM constant; the subscript 0 indicates the total concentration. When the substrate concentration ($[S]_0$) is equal to K_M, the initial velocity reaches $1/2 \cdot v_{max}$. The k_{cat} term corresponds to the rate-limiting step in enzymatic reactions, thus playing a critical role in the overall catalytic efficiency of the enzyme.

$$\equiv \frac{d[P]}{dt} = k_{cat}[ES] - k_{-cat}[E][P]$$

Dissociating the *ES* complex is often considered an irreversible process because of the low affinity of the enzyme to the product. As a result, k_{-cat} is negligibly small.[8]

$$\approx k_{cat}[ES] \ or \ v_{max} = k_{cat}[E]_0$$

Experimentally, the fluorogenic substrate was diluted in Tris-HCl buffer (20 mM, pH 7.4) to reach final substrate concentrations of 0, 1, 3, 6, 12, 18, 24, 30, 36 µM in a 60 µL volume within a 96-well plate. The alpha-thrombin ($[E]_0$ = 20 nM to a 60 µL volume) was then added to each well and the total volume was brought to 60 µL. Next, the 96-well plate was incubated at 37 °C in a hybrid multi-mode microplate reader with 10 s of shaking before each cycle of readout. The PL intensity at 570 nm for Cy3 was recorded over 12 h with 1 min intervals between each cycle.

At least three replicates were performed, and the results were averaged and plotted against substrate concentrations. Error bars indicate the standard deviation of the means. The $\Delta PL = PL_{30min} - PL_{0min}$ was correlated to the product concentration using a standard curve: ΔPL_{Cy3} *versus* full-digested FRET probe. The data were fitted following the Michaelis-Menten equation.[9]

2.9. Specificity test

Bovine serum albumin (BSA, M_w: 66463 Da), hemoglobin (M_w: 64500 Da), alpha-thrombin (M_w: 36,000 Da), α-amylase (1000 U/mL), and M^{pro} (Main protease of SAR-CoV-2) were used for the specificity test. Briefly, the desired amounts of BSA, hemoglobin, alpha-thrombin, α-amylase, M^{pro}, and human saliva were spiked into Tris-HCl buffer (20 mM, pH 8.5) to reach the final concentration of 1 μM. Then, 100 μL of nano-coacervates (c_{final} = 1 mM) were mixed with different enzymes. The PL signal of the mixture at 670 nm was read at 37 ºC every 1 min for 1 h. The fluorescence spectra from 660 nm and 900 nm were measured after a 1 h incubation. The wavelength for excitation was 630 nm. At least three replicates of each experiment were performed to measure the average and standard deviation.

2.10. Heparin release confirmation

Briefly, nano-coacervates were incubated with and without 1 μM of thrombin to disassemble nano-coacervate. After centrifugation at 3 x g for 10 min, the supernatant was collected, and the released heparin of 0.2, 0.4, 0.6, 0.8, 1, 1.2, and 1.4 U/ml was incubated with 20 μL of MB dye (100 μM) shown in Figure 2i and Supplementary Figure 17. In parallel, the same concentration of heparin fresh from the vendor was incubated with 20 μL of MB dye to compare a decrease in absorbance at 666 nm. The supernatant collected from nano-coacervates without thrombin was examined as a negative control.

2.11. Stability test of NC-$TA_{0.13}$ in different media

The tannic-acid encapsulated nano-coacervates (NC-$TA_{0.13}$) of 1 mM were incubated in different conditions of 50% acetone, methanol (MeOH), citric acid (pH 2), DPBS, NaOH, 60 °C, NaCl of 150 mM, 50% of human urine, saliva, and DMEM, respectively. We also tested coacervate stability using glutamine, glucose (5.6 mM), human albumin (0.6 mM), and fibrinogen (8.8 μM). The glucose[10], albumin[11], and fibrinogen[12] concentrations fall in physiological conditions. After 3h incubation, the samples were centrifuged at 3 x g to replace the solvent condition with MQ water. Then, the hydrodynamic diameter of NC-$TA_{0.13}$ was measured using a Malvern Instrument Zetasizer ZS 90.

2.12. ELISA test to measure F1+2 peptide

Human blood specimens were collected under approval from the institutional review board (IRB) of UC San Diego and the UCSD VA (#H170005). All subjects gave written informed consent. All work was done in accordance with the Declaration of Helsinki. Briefly, 400 μL of human blood was incubated with heparin, NC-$TA_{0.13}$, scramble NC-$TA_{0.13}$, tannic acid, and C5 peptides for 15 min and centrifuged at 1 x g for 30 min to collect human plasma or serum for enzyme-linked immunoassay (ELISA) test. Heparin was titrated to measure the prevention point of blood coagulation (Supplementary Figure 35). The fresh blood was drawn using an EDTA-treated

blood collection tube. Calcium chloride was used to activate blood coagulation by EDTA-Ca^{2+} chelation (Figure 4i and Supplementary Figure 35). The experiments were independently performed twice and obtained similar results.

In addition, blood collection tubes containing 200 µL of PBS (–), heparin, NC, and NC-coacervates were directly used to collect 2 ml of human blood (Supplementary Figure 36). After 15 min incubation, the sample was centrifuged at 1 x g for 30 min to collect human plasma or serum. The final concentrations of heparin were 40 U/ml in this test. The sample was centrifuged until no precipitates appeared, and the supernatant was collected for the ELISA test.

After all the sample collection, 100 µL of each diluted sample was transferred to a test sample well. The microplate was sealed with a plate sealer and incubated at 37 °C for 1.5 h. Following the procedure described in the protocol (Abbexa, abx252419, UK), the samples were treated with reagents. Finally, TMB solvent was used to activate the enzyme. For F1+2 peptide calculation, averaged O.D. at 450 nm readings for each reference standard and each sample was subtracted with the averaged control (zero) O.D. readings.[13]

(Relative *O.D.* at 450 nm) = (*O.D.* of Each Well) – (*O.D.* of Zero Well)

2.13. Turbidity calculation

Turbidity measurements were calibrated using a multi-mode microplate reader (Synergy™ H1 model, Bioteck) in a 96-well plate. The extinction (*i.e.,* absorbance) at 500 nm of coacervate samples (peptide-heparin complex) was measured after inducing complex coacervation. Turbidity value was measured with the following equation:[1]

$$Turbidity = 100 - 10^{(2 - extinction_{500\,nm})} = 100 - \%T$$

2.14. Cytotoxicity Test and Cell Staining of HEK 293

HEK293 (ATCC, CRL-1573) cells were cultured in Dulbecco Modified Eagle Medium (DMEM) medium with 10% fetal bovine serum. Cell cultures were incubated under 5% CO_2 at 37 °C. Cells were passaged when they reached 75 to 80% confluency using 0.25% trypsin-EDTA. DPBS and CTAB were used for negative and positive controls of healthy and dead cells. For the experiments, HEK 293 cells were seeded overnight in a 96-well plate at a concentration of 10,000 cells/well. Subsequently, PBS, CTAB, nano-coacervates, NC-$TA_{0.13}$, NC-$TA_{0.33}$, and NC-TA_1 were co-incubated with HEK 293 cells at an equal concentration of 0.16 mM for 12 h. A

resazurin assay was used to analyze the cytotoxicity of nano-coacervates, NC-$TA_{0.13}$, NC-$TA_{0.33}$, and NC-TA_1 following a protocol. After 4 h incubation with resazurin, cell viability was calibrated by measuring the subtracted background absorbance of each well at 600 nm from resazurin absorbance at 570 nm. The absorbances of experimental wells were compared to those of the controlled wells containing healthy and dead cells. For cell staining, Hoechst and propidium iodide (PI) were used to stain cell nuclei and dead cells. HEK 293 cells were seeded in a 12-well plate (50,000 cells/well) overnight and then incubated with nano-coacervates, NC-$TA_{0.13}$, NC-$TA_{0.33}$, and NC-TA_1 with the same concentration of 0.16 mM for 24 h. After incubation, a mixture solution of Hoechst and PI was added to stain the HEK 293. The fluorescence images of each sample were obtained using an EVOS FL fluorescence microscope after gently washing with PBS.

2.15. General characterizations

1. Transmission electron microscopy (TEM) and energy dispersive X-ray (EDX) images were collected using a JEOL JEM-1400 Plus operating at 80 kV in the Nano3 cleanroom UCSD. Gatan 4k digital camera with installed software was used to process TEM images. Tomography TEM and EDX images with different angles were collected using an advanced tomography holder. 2 μL of each diluted sample was dropped on the carbon grids and dried. Subsequently, uranium staining was performed under a fume hood to stain the peptide samples. A single drop of diluted 0.1% uranium solution was placed on the TEM grid for 30 s. After that, the TEM grid was washed with MQ water three times independently and dried for the measurement.
2. The hydrodynamic diameter was calibrated based on dynamic light scattering (DLS) using a Malvern Instrument Zetasizer ZS 90. Likewise, the zeta potential (i.e., surface charge) of the sample was measured using a Malvern Instrument Zetasizer ZS 90.
3. Absorbance spectra were measured using a BioTek Synergy H1 plate reader. Each sample was measured in 96-well plates. Absorbance was collected from 300 to 900 nm with a step size of 2 nm. Photoluminescence (PL) of fluorescent dyes or peptide-dye conjugates (C7 and C8) was measured using a desired excitation and emission wavelength: 675/695 nm for sulfo-Cy5.5 and 555/570 nm for Cy3. A seal film was attached to the 96-well plate to prevent solvent evaporation when performing time-dependent measurements. Baseline correction (i.e., solvent only) was performed to subtract the background signal.
4. Fourier transform infrared (FTIR) data was obtained using A Bruker Tensor II FTIR spectrophotometer. The samples were dried using a lyophilizer, and the dried sample was used for the measurement.
5. Multi-laser nanoparticle tracking analysis (M-NTA) measurement was performed using a ViewSizer 3000 (Horiba Scientific, CA, USA) to measure particle concentration, sample dispersion, and size.[14] Ten videos (8-bit) were recorded with 300 frames for seconds. Particle size was measured using the recorded 10 videos through imaging analysis installed

in the instrument. A quartz cuvette with a minimum sample volume of 0.8 mL was used for the measurement. Each sample was diluted in 5 mL of DI water to prevent signal saturation.

6. The desired peptides were confirmed using electrospray ionization mass spectrometry (ESI-MS) *via* the Micromass Quattro Ultima Mass Spectrometer in the Molecular Mass Facility (MMSF) at Chemistry and Biochemistry Department at UC San Diego. The sample for ESI-MS was prepared using a 50% MeOH/H_2O mixture. 2 μL of each sample was injected into the instrument independently three times to collect ESI-MS data.
7. The desired peptides were confirmed using a matrix-assisted laser absorption ionization-time of flight mass spectrometry (MALDI-TOF MS, Bruker Autoflex Max) in the MMSF at UC San Diego. Each sample was mixed with an HCCA matrix (1:3 ratio of sample to the matrix). Subsequently, 1-2 μL of the mixture was placed on the plate and dried using a heat gun. The measurement was repeated three times to confirm the mass peaks.
8. Scanning electron microscopy (SEM) images were taken using Zeiss Merlin. The coacervate sample was dropped on the silicon wafer and dried overnight. 10 nm of gold was coated on the coacervates through sputtering to improve image resolution.
9. Thrombin chromogenic substrate was used to determine thrombin activity. 10 mM of stock solution was prepared using 1 mM HCl and diluted 1:50 in PBS, producing 0.2 mM working solution. 20 μL test sample was incubated with 180 μL substrate and monitored color change at 405 nm.
10. Confocal images were obtained using a Leica STED Sp8 with Falcon microscope (Wetzlar, Germany) with an x63 oil objective at room temperature. The sample was prepared in a Lumox 96-well cell culture plate for confocal imaging. The excitation and emission wavelengths were 405 nm and 435-600 nm, respectively.

3. Supplementary Notes: Thrombin

Thrombin[15] is a key enzyme in haemostasias and plays an important role in the body's response to blood vessel or tissue injuries. When a blood vessel is damaged, there is an immediate surge of thrombin. This prompts the rapid formation of a plug comprised of platelets and fibrin and triggers the blood clotting process. Thrombin exists in plasma as an inactive precursor protein: prothrombin (*i.e.*, blood clotting factor II). Tissue damage triggers the activation of factor VII which sets off a cascade: Factor VIIa activates factor X, which in turn activates prothrombin amplifying the clotting process. Factor Xa can also significantly accelerate the activation of prothrombin (by a factor of 1000-fold). The result of the thrombin generation is the clotting of fibrinogen. Thrombin cleaves two fibrinopeptides (A and B) from fibrinogen, transforming it into fibrin monomers. These monomers then spontaneously polymerize into elongated fibrin stands. Additionally, thrombin activates another enzyme factor XIII, an enzyme that crosslinks fibrin monomers to establish a stable and robust fibrin network. Consequently, this network facilitates wound closure.[15] Alpha-thrombin is composed of a light chain (A chain, $M_w \sim$ 6kDa) and a heavy chain (B chain, M_w ~31kDa). These two chains are coupled by one disulfide bond.

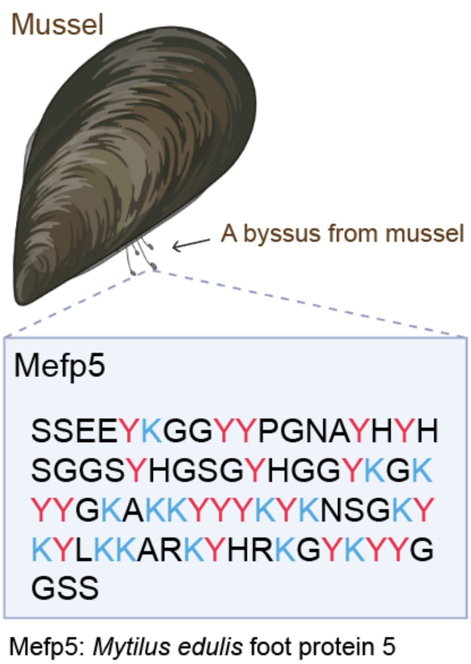

Supplementary Figure 1: Schematic of mussel foot protein
Mytilus edulis foot protein 5 found from a byssus of mussel is made of repeating units of DOPA and lysine (K) amino acids which provide strong wet adhesive properties through hydrophobic and positive charge.[16] This panel created with BioRender.com released under a Creative Commons Attribution-NonCommercial-NoDerivs 4.0 International license.

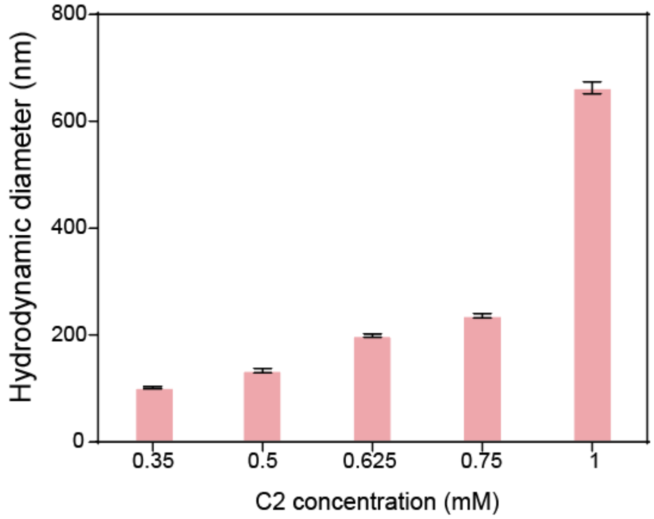

Supplementary Figure 2: Different-sized coacervate droplets

C2 peptides at different concentrations of 0.35, 0.5, 0.625, 0.75, and 1 mM were used to induce coacervates at a fixed concentration of heparin (50 U/ml). The average hydrodynamic diameters of 0.35, 0.5, 0.625, 0.75 and 1 mM were 101.3 ± 2.1 nm, 133.2 ± 3.8 nm, 198.5 ± 3.3 nm, 235.9 ± 4.0 nm, and 662.2 ± 11.0 nm, respectively. Nano-coacervates (sizes below 300 nm) maintained their colloidal stability and size while coacervates with a size over 500 nm continuously grew and became micro-sized coacervates. Data represent mean ± SD (n = 3).

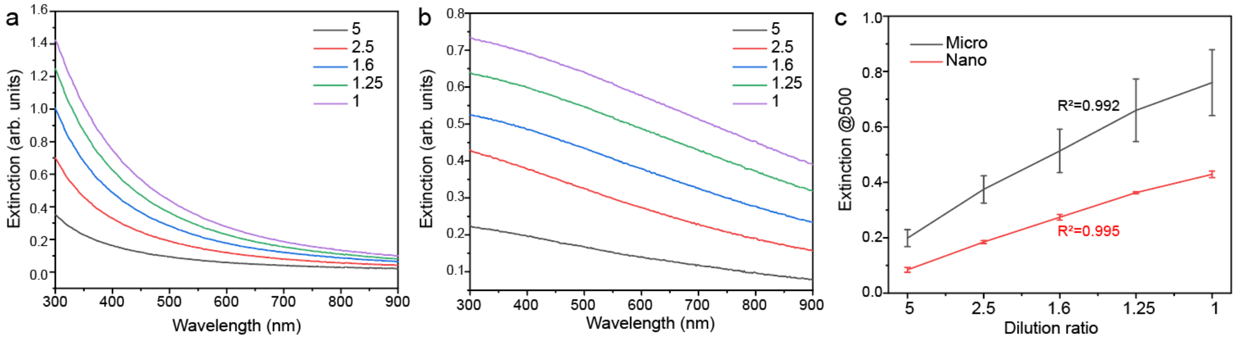

Supplementary Figure 3: UV-vis spectra of nano- and micro coacervates

UV-vis spectra of nano- (a) and micro- (b) coacervates in different dilution ratios: the ratio of the sample to the solvent was 1 to 1, 1.25, 1.6, 2.5, and 5, respectively. c) Extinction (i.e., absorbance) at 500 nm of nano- and micro-coacervates are dependent on particle concentration explained by Beer's law. Data represent mean ± SD (n = 3).

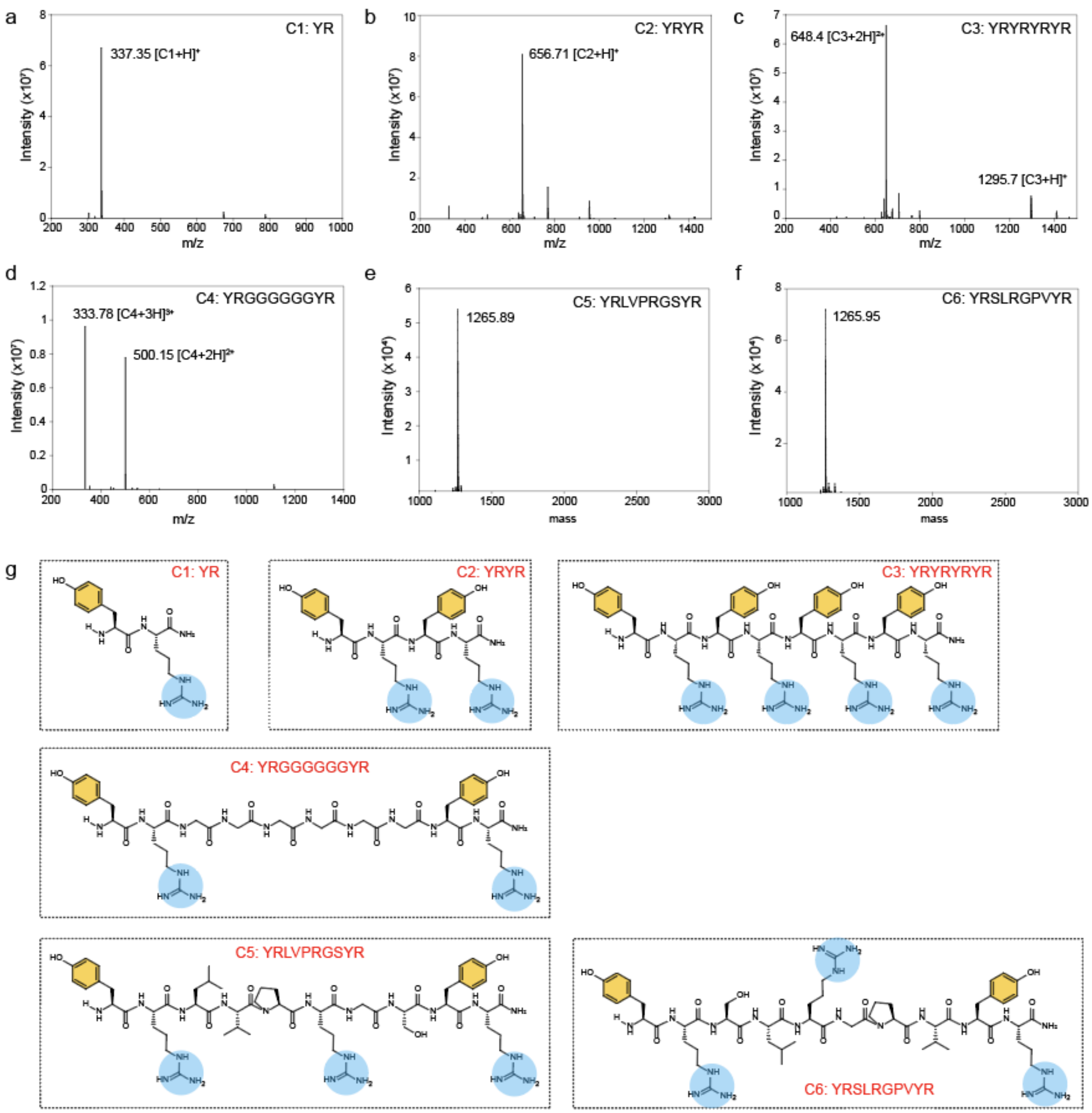

Supplementary Figure 4: Mass peaks of C1–C6 peptides

ESI-MS data of C1 (a), C2 (b), C3 (c), and C4 (d) as well as MALDI-TOF data of C5 (e) and C6 (f) peptide sequences. g) Chemical structure of C1, C2, C3, C4, C5, and C6 sequence, respectively. The yellow ring and blue area indicate tyrosine (Y) and arginine (R) peptides that provide hydrophobicity and positive charges to interact with heparin.

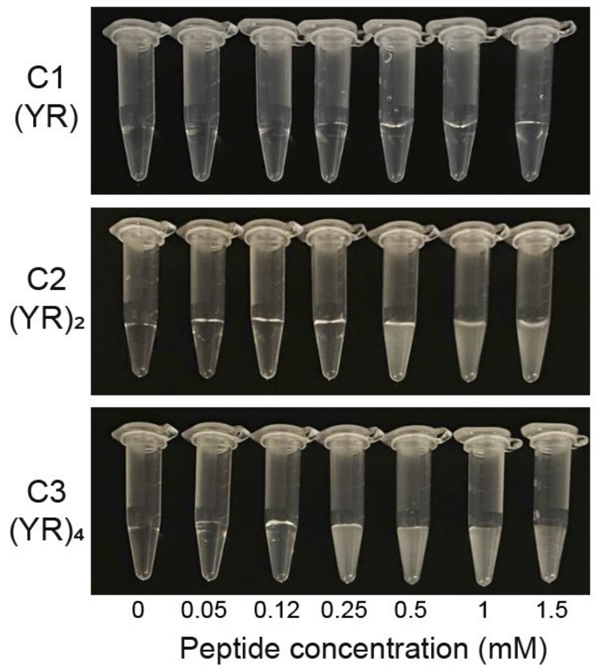

Supplementary Figure 5: Photographs of C1, C2, and C3- based coacervates
C2 and C3 peptides with different concentrations ranging from 0.05 to 1.5 mM were combined with a constant heparin concentration of 50 U/ml, thus forming coacervate droplets. In contrast, there was no formation of coacervates when C1 peptides were mixed with heparin. The photographs show that C2 and C3 peptides became turbid as a function of coacervate formation, but there was no turbidity change in C1-heparin mixtures (i.e., transparent).

a

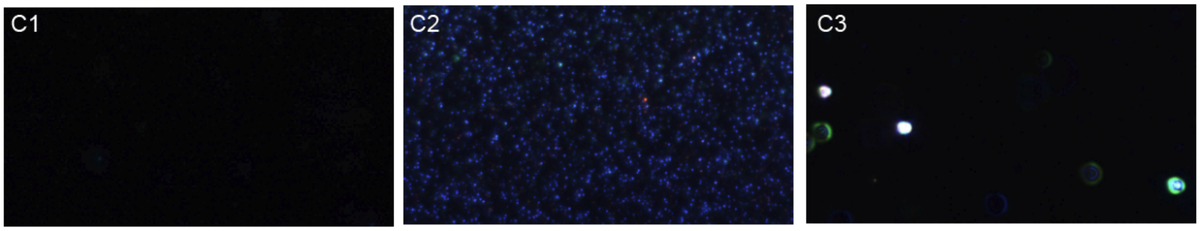

b

Results	
Total frames / Processed frames	2100 / 2100
Binning	Logarithmic binning, max 20 um
Integration range	100 [nm] to 1900 [nm]
Counts within range* / Total tracks*	1568 / 2310
Concentration within range	8.8E+07 [particles/mL]
Diluent sample name	-
Diluent concentration within range	-
Concentration with diluent subtracted	8.8E+07 [particles/mL]
Average size within range of integration	145 [nm]
Standard deviation of size / CV	43 [nm] / 0.29
Modal size	98 [nm]
D10 / D50 / D90*	66.12 [nm] / 116.96 [nm] / 172.80 [nm]
Average viscosity during measurement	0.98 [cP]

*computed with ConstantBins table

c

Results	
Total frames / Processed frames	2100 / 2100
Binning	Logarithmic binning, max 20 um
Integration range	100 [nm] to 1900 [nm]
Counts within range* / Total tracks*	47 / 76
Concentration within range	2.6E+06 [particles/mL]
Diluent sample name	-
Diluent concentration within range	-
Concentration with diluent subtracted	2.6E+06 [particles/mL]
Average size within range of integration	685 [nm]
Standard deviation of size / CV	429 [nm] / 0.63
Modal size	14 [nm]
D10 / D50 / D90*	33.03 [nm] / 290.50 [nm] / 1139.90 [nm]
Average viscosity during measurement	0.99 [cP]

*computed with ConstantBins table

Supplementary Figure 6: M-NTA data of C1, C2, and C3-based coacervation
M-NTA images of C1(a), C2 (b), and C3 (c)-based coacervates. C2 and C3 peptides formed nano- and micro-sized coacervates, respectively, while C1 peptides failed to form coacervates. M-NTA size profile of nano- (b) and micro- (c) coacervates. The red box indicates the average size and standard deviation measured by M-NTA. Supplementary Movies 1-3 show C1, C2, and C3-based coacervate droplets (details described on page 51).

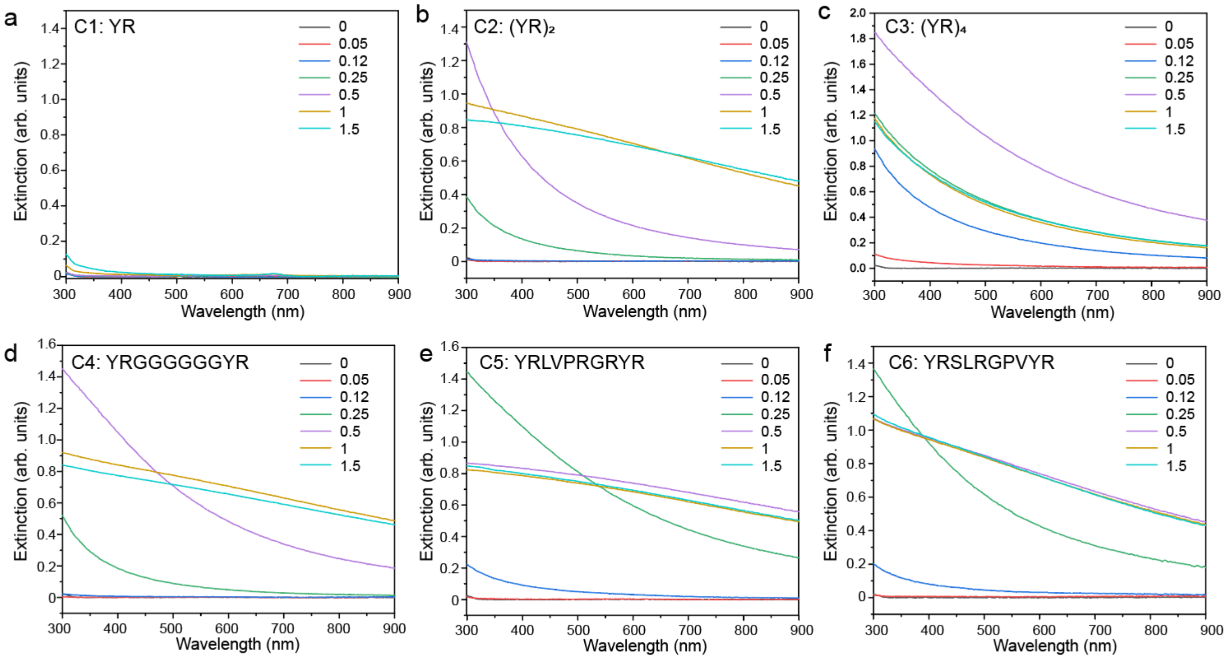

Supplementary Figure 7: UV-vis spectra of C1–C6 based coacervates

Different peptide sequences including C1 (a), C2 (b), C3 (c), C4 (d), C5 (e), and C6 (f) were examined to form coacervates with fixed heparin concentration of 50 U/ml. The results showed that the number of YR is important to form nano- or micro-coacervates: The single YR unit failed to form coacervates due to a lack of charge and hydrophobicity. Extinction spectra of nano-coacervates increased from 900 to 300 nm while micro-coacervates showed a broad extinction spectrum likely due to increased light scattering. In addition, C4-based coacervation showed similar extinction spectra as C2-based coacervation. Likewise, C5 and C6 peptides which have the same number of YR units showed similar extinction spectra.

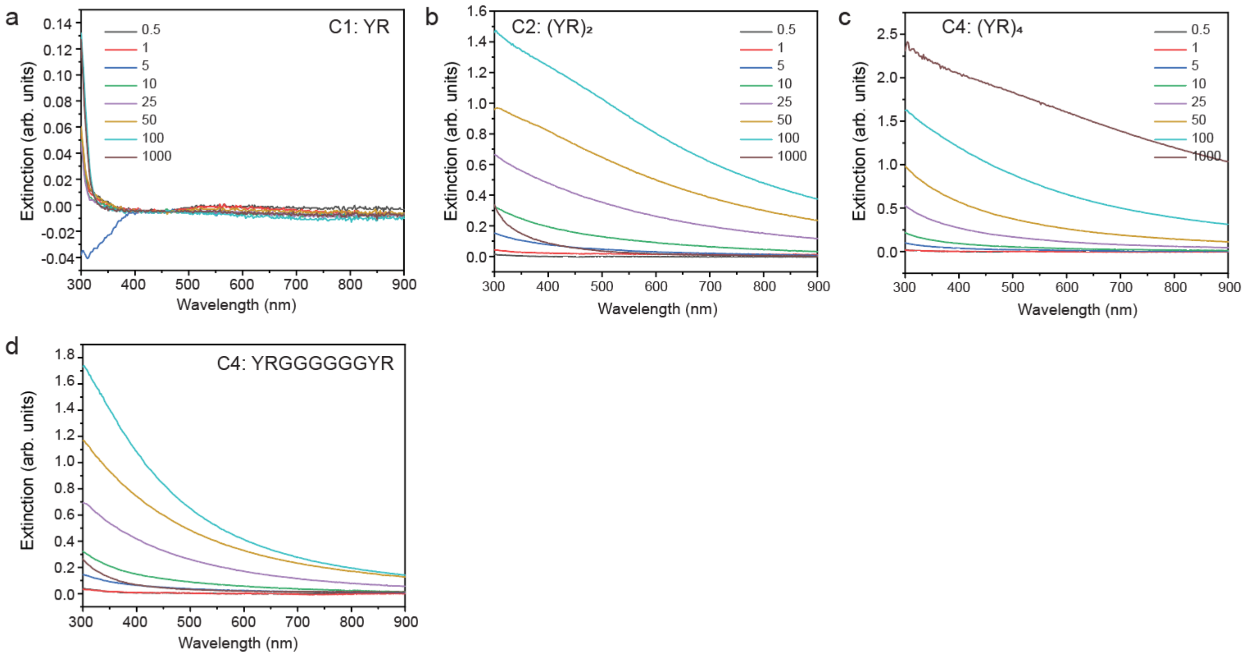

Supplementary Figure 8: UV-vis spectra of C1–C4-based coacervates with the fixed peptide concentrations

UV-vis spectra of C1 (a), C2 (b), C3 (c), and C4 (d) coacervate formation with various heparin concentrations ranging from 0.5 to 1000 U/ml. The peptide concentration was constant (1 mM). The C2, C3, and C4 peptides could form nano- or micro-sized coacervates with heparin.

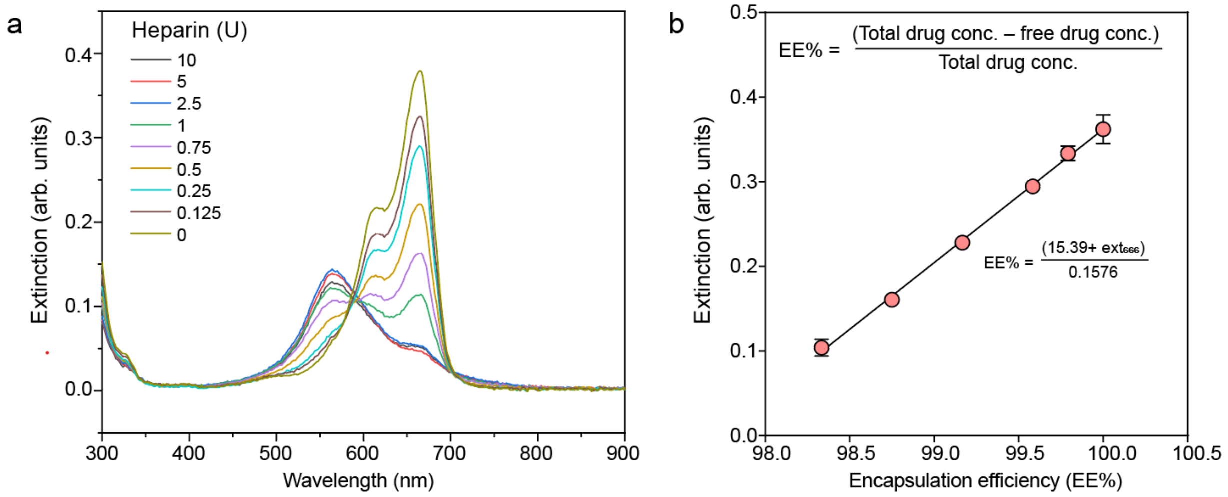

Supplementary Figure 9: Calculation of heparin encapsulation efficiency
a) UV-vis spectra of MB-heparin complex. MB dyes were mixed with different concentrations of heparin-inducing MB-heparin aggregates that in turn decreased the absorption peak to 666 nm and increased the peak to 580 nm. b) Encapsulation efficiency formula was built using the decreased extinction value at 666 nm.[17] Data represent mean ± SD (n = 3).

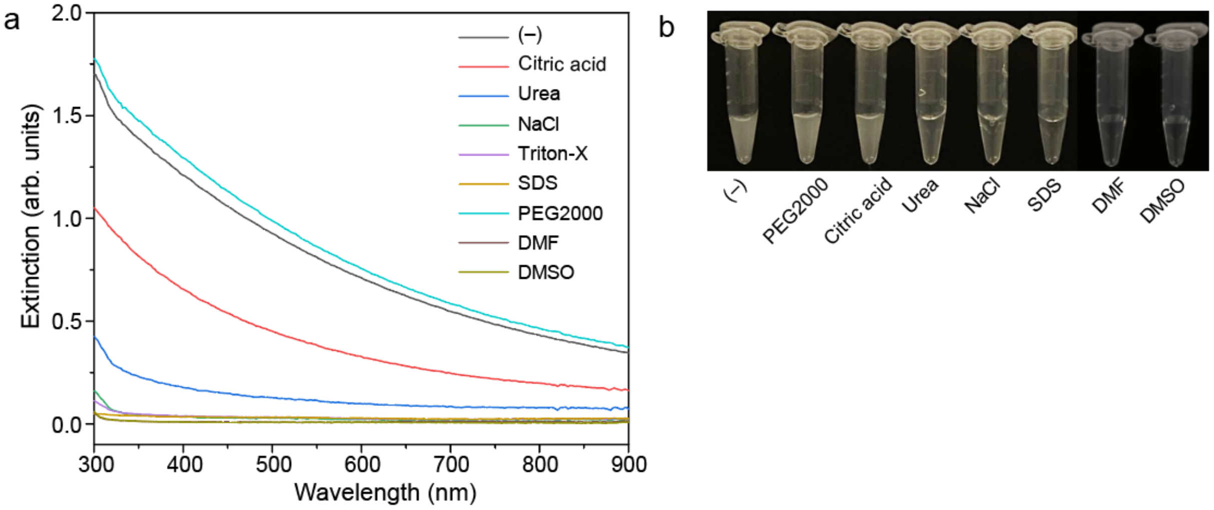

Supplementary Figure 10: Stability test of nano-coacervates in different environments
Nano-coacervates (average size: 200 nm) at the same concentration of 1 mM were incubated in various conditions including MQ water (as a negative control), PEG2000 (10 mg/ml), citric acid (pH 2), 10 mM of urea, NaCl, SDS (10 mg/ml), 80% of DMF, and DMSO, respectively. a) UV-vis spectra illustrated a decrease in the extinction of nano-coacervates because of the disassembly of nano-coacervates, confirming internal interactions for coacervation.[2] b) Photograph of nano-coacervates in MQ water, PEG2000, citric acid, urea, NaCl, SDS, DMF, and DMSO, respectively.

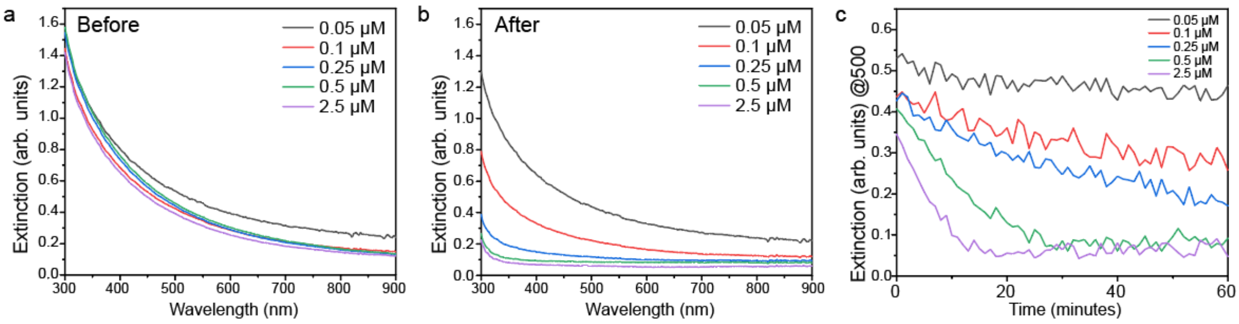

Supplementary Figure 11: Turbidity changes at different thrombin concentrations

UV-vis spectra of nano-coacervates before (a) and after (b) thrombin incubation. c) Time-dependent changes in extinction at 500 nm. Nano-coacervates (1 mM) were incubated with various concentrations of thrombin, ranging from 0.05 to 2.5 μM in 20 mM Tris-HCl buffer (pH 8.5) at 37 °C for 1 h. The results indicate that higher thrombin concentrations induce a rapid disassembly of the nano-coacervates. The C5 peptide was used for the formation of nano-coacervates.

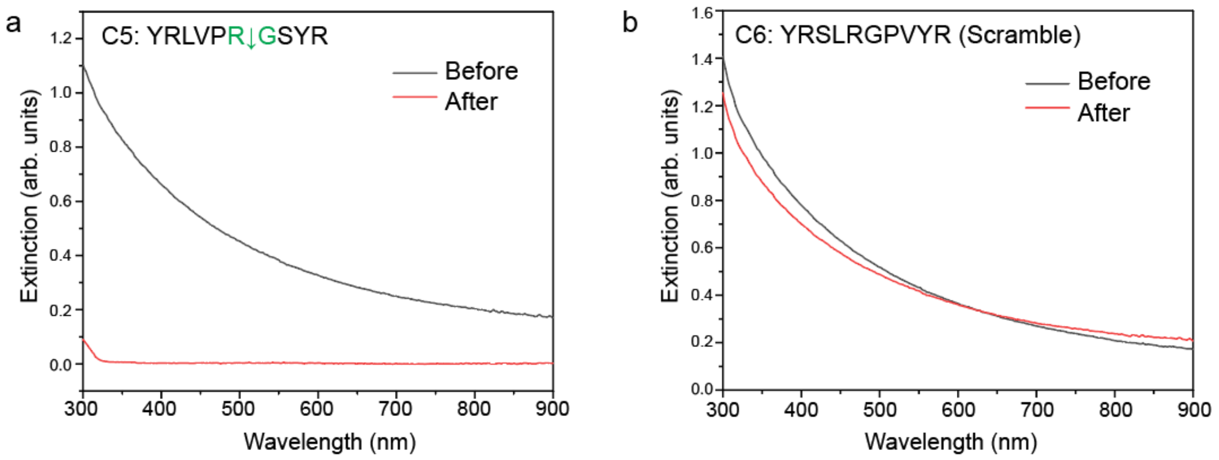

Supplementary Figure 12: Thrombin proteolytic test on nano-coacervates composed of scramble sequence

UV-vis spectra of C5 (a) and C6 (b) based nano coacervates before (black) and after (red) thrombin incubation. The C5- and C6-based nano-coacervates at the same concentration of 1 mM were incubated with 500 nM of α-thrombin at 37 °C for 1 h. The results showed that thrombin was incapable of disassembling the nano-coacervates composed of a scramble sequence (i.e., C6).

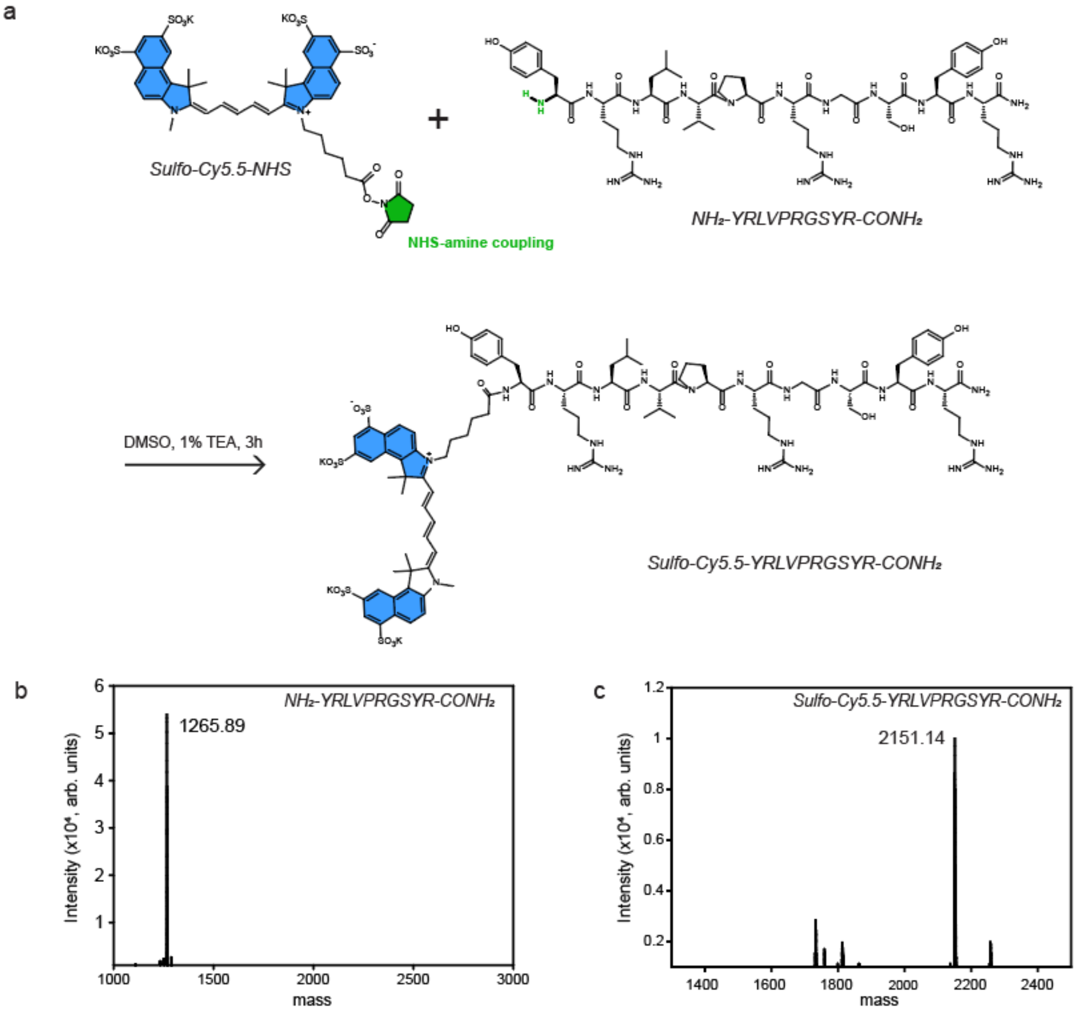

Supplementary Figure 13: Sulfo-Cy5.5 conjugation to C5 peptide (i.e., C7)

a) Schematic illustration of NHS-amine coupling of sulfo-Cy5.5-NHS and C5 peptide: NH_2-YRLVPRGSYR-$CONH_2$. MALDI-TOF data before (b) and after (c) sulfo-Cy5.5 conjugation, confirming that sulfo-Cy5.5 dyes were successfully conjugated to C5 peptide. Sulfo-Cy5.5 needs coupling with C5 peptide to be encapsulated within the nano-coacervates. Without conjugation with the C5 peptide, sulfo-Cy5.5 was not incorporated within the nano-coacervates. A negatively charged sulfonate group on Cy5.5 is required to activate the fluorescent signal upon disassembly of the nano-coacervates. Without the sulfonate group, the positively charged Cy5.5 dyes strongly interacted with heparin, quenching its fluorescence even after disassembly. Note that sulfo-Cy5.5 without C5 peptide did not encapsulate into the nano-coacervates.

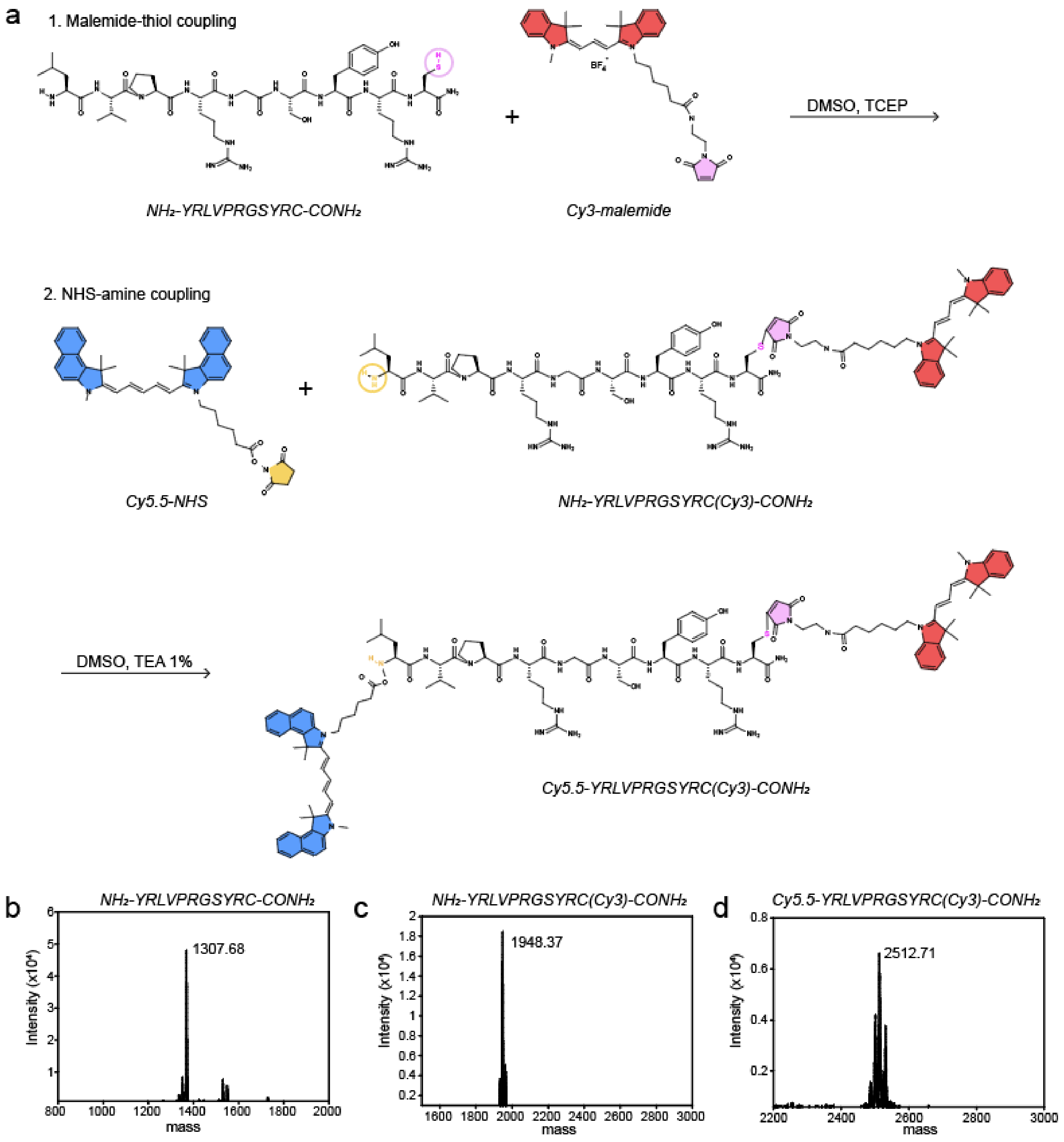

Supplementary Figure 14: Cy5.5-YRLVPRGSYRC(Cy3) preparation (*i.e.*, C8)

a) Schematic illustration of maleimide-thiol and NHS-amide couplings to prepare Cy5.5-YRLVPRGSYRC-Cy3 (i.e., C8) for k_{cat}/K_M measurement. MALDI-MOF data of peptide only (b), peptide-Cy3 (c), and Cy5.5-peptide-Cy3 (d). The peptide sequence used for dye-conjugation is NH_2-YRLVPRGSYRC-$CONH_2$.

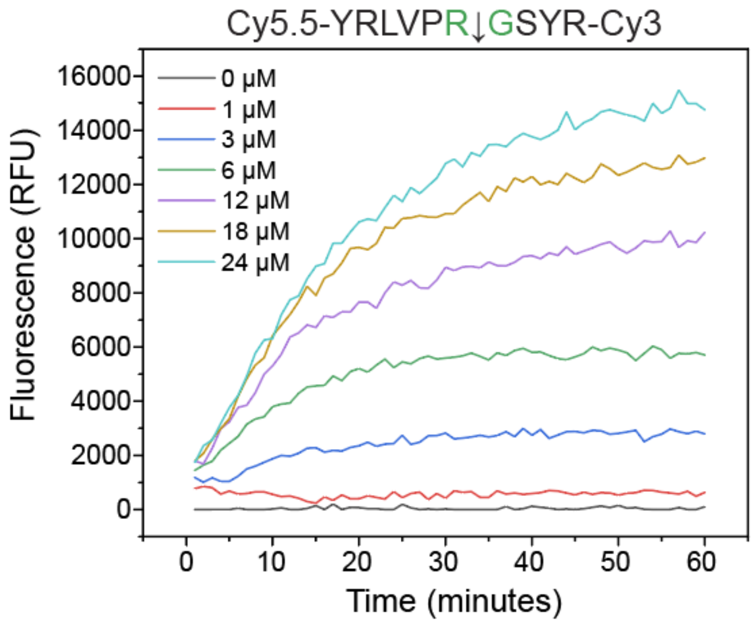

Supplementary Figure 15: k_{cat}/K_M measurement

Time-dependent Cy3 fluorescence activation driven by thrombin proteolysis. Fluorogenic substrate (Cy5.5-YRLVPRGSYRC(Cy3), C8 peptide) with different concentrations from 1 to 24 µM was incubated with 20 nM of enzyme (i.e., α-thrombin). At least three replicates were performed to measure mean and standard deviations.

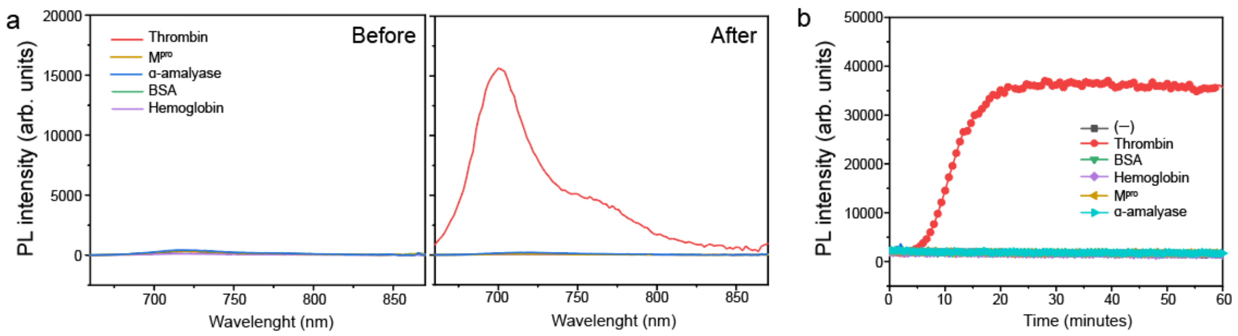

Supplementary Figure 16: Specificity test with different enzymes

a) PL intensity of the C7-encapsulated nano-coacervates before and after enzyme incubation. Different enzymes including thrombin, bovine serum albumin (BSA), hemoglobin, main protease of SARS-CoV-2, and α-amylase at the same concentration of 5 μM were incubated with nano-coacervates, respectively. The C7-encapsulated nano-coacervates activated the PL signal upon the disassembly of the nano-coacervates. b) Time-dependent signal activation induced by different enzymes, showing that thrombin can activate PL signals.

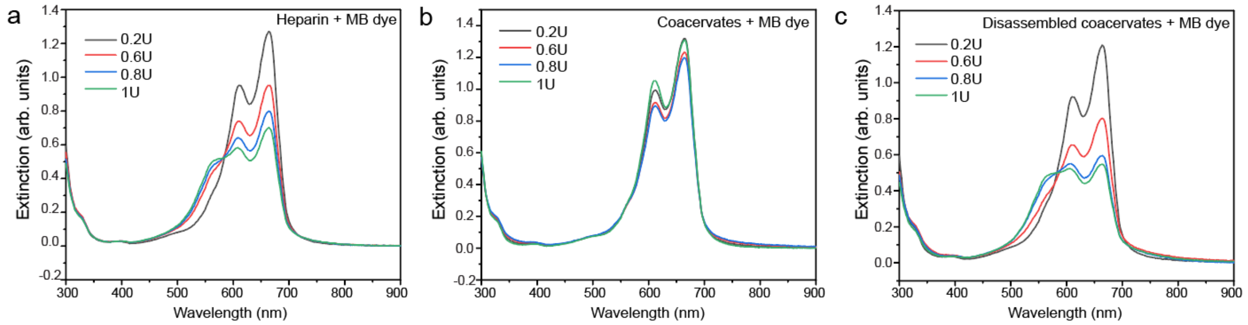

Supplementary Figure 17: Heparin release confirmed by the formation of MB-Heparin complex

a) UV-vis spectra of the MB-heparin complex. The positively charged MB dye can form aggregates with the negatively charged heparin, thus reducing the absorption peak at 666 nm. UV-vis spectra of nano-coacervates mixed with the MB dye before (b) and after (c) the addition of thrombin. The release of heparin due to thrombin cleavage resulted in a decrease in absorbance at 666 nm, whereas intact nano-coacervates exhibited no change in absorbance. 60 μL of the supernatant was incubated with 20 μL of MB dye (100 μM) for absorbance measurement.

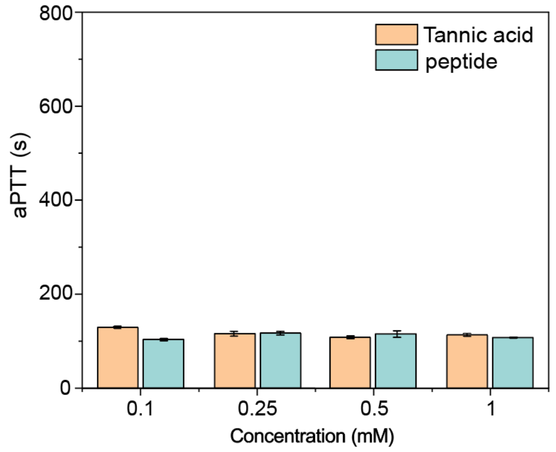

Supplementary Figure 18: aPTT test of C5 peptide and tannic acid
Plasma coagulation was observed in aPTT tests using C5 peptide and tannic acid with different concentrations from 0.1 to 1 mM. Data represent mean ± SD (n = 3).

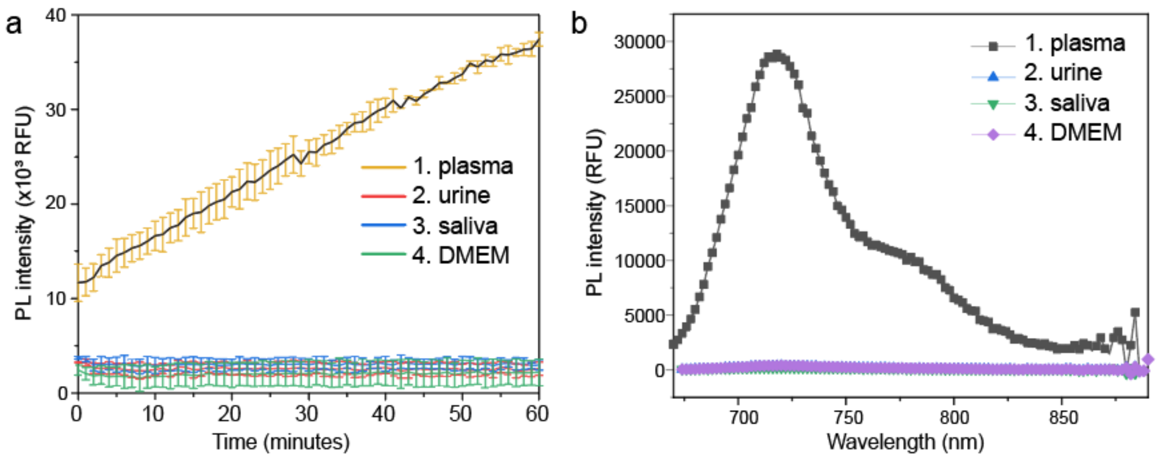

Supplementary Figure 19: Colloidal stability of nano-coacervates in biofluids

C7-encapsulated nano-coacervates were incubated in various biofluids, including 50% human plasma, urine, saliva, and DMEM, respectively. a) Time-dependent PL intensity of the nano-coacervates containing C7 peptides. Disassembly of the nano-coacervates led to an increase in fluorescence. b) Nano-coacervates were unstable in human plasma, resulting in the disassembly of coacervate phases and subsequently activated PL intensity. However, the nano-coacervates incubated in human urine, saliva, and DMEM showed no PL activation, indicating its stability. Data represent mean ± SD (n = 3).

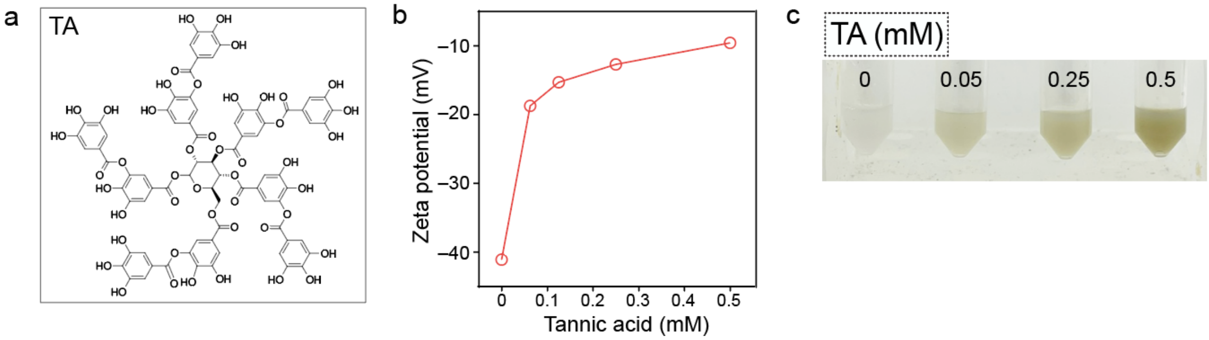

Supplementary Figure 20: Photograph and surface charge of NC-TAs

a) Chemical structure of TA molecule. b) The surface charge of nano-coacervates, NC-TA$_{0.05}$, NC-TA$_{0.25}$, and NC-TA$_{0.5}$. The zeta potential (*i.e.,* surface charge) of nano-coacervates, NC-TA$_{0.05}$, NC-TA$_{0.25}$, and NC-TA$_{0.5}$ were –41.1 ± 0.4 mV, –18.7 ± 0.1 mV, –15.3 ± 0.6 mV, and –12.6 ± 0.4 mV, respectively. c) Photograph of nano-coacervates, NC-TA$_{0.05}$, NC-TA$_{0.25}$, and NC-TA$_{0.5}$. The color of the solvent changed from turbid white to yellowish brown after TA encapsulation.

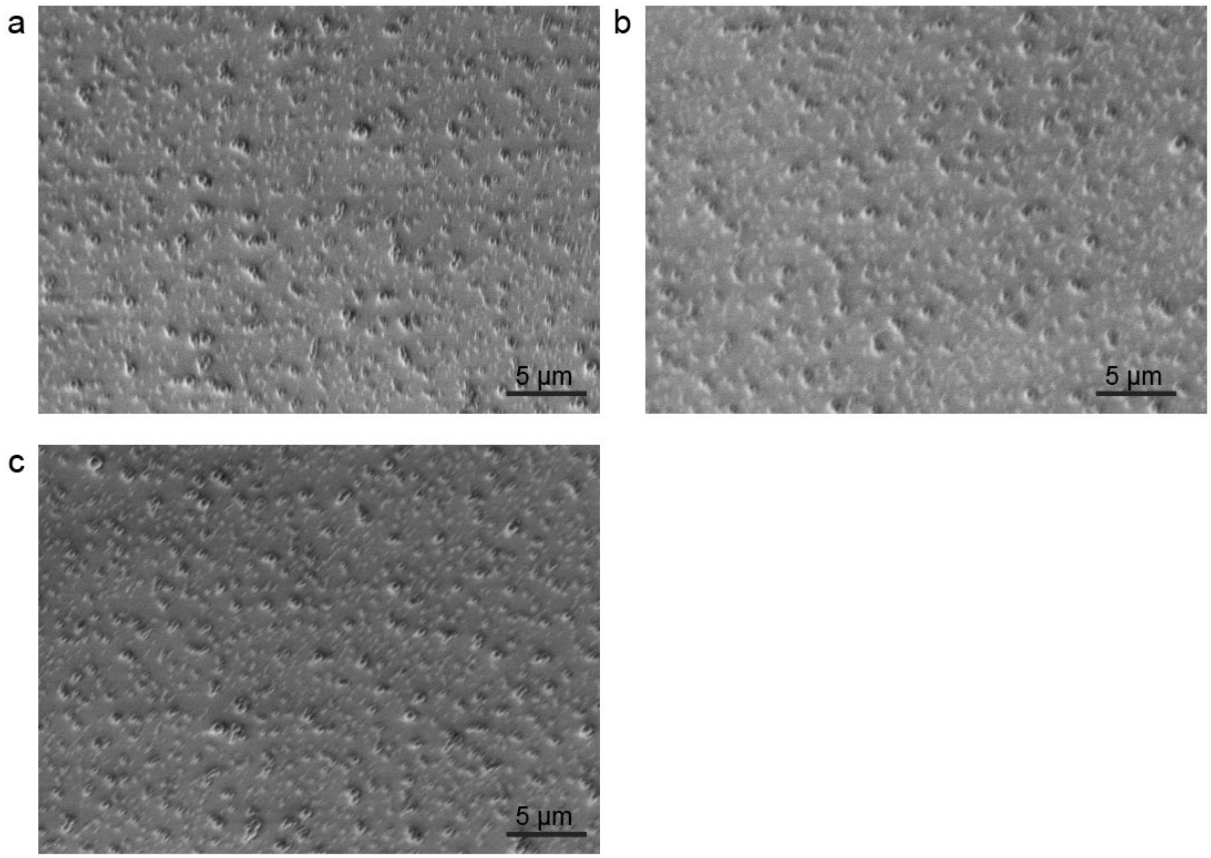

Supplementary Figure 21: SEM image of NC-TAs
SEM images clearly visualized the mono-dispersed NC-$TA_{0.13}$ (a), NC-$TA_{0.33}$ (b), and NC-TA_1 (c) dried on the silicon wafer.

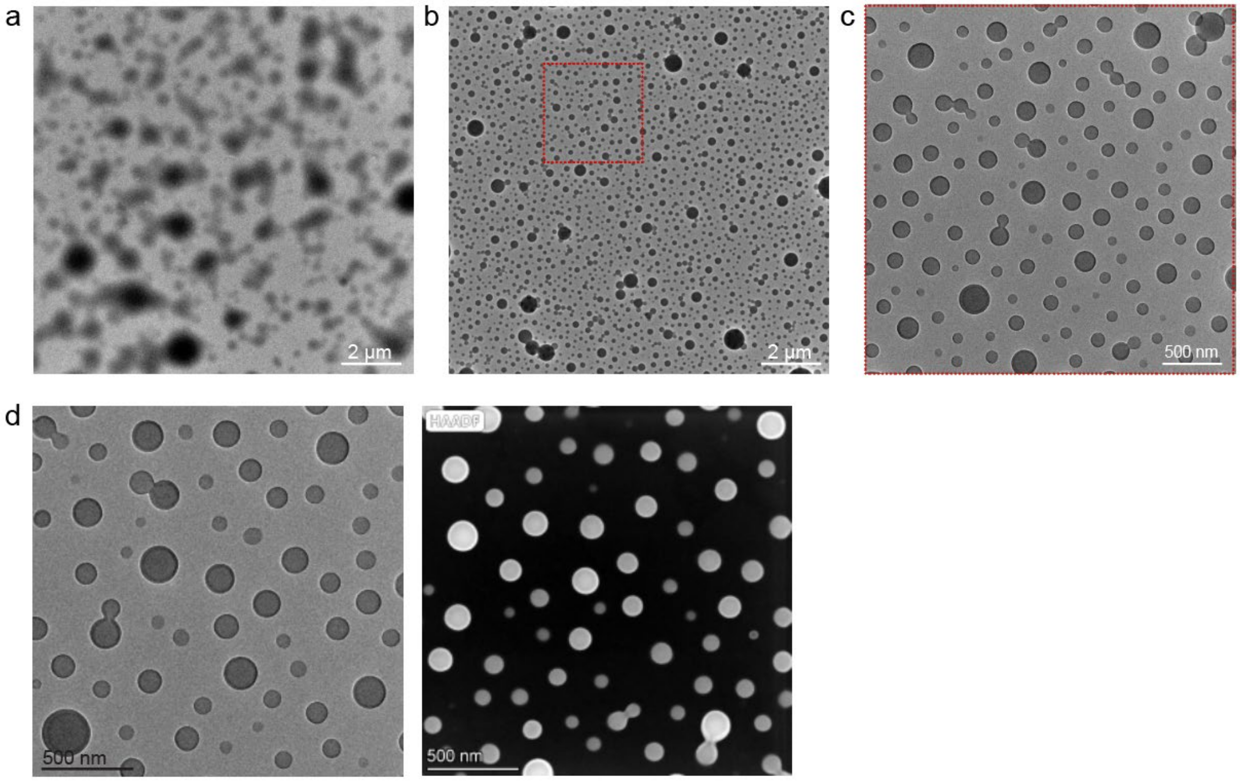

Supplementary Figure 22: TEM images of nano-coacervates and NC-TAs
TEM images of nano-coacervates (a) and NC-$TA_{0.13}$ (b) with low magnification. Panel a indicates that nano-coacervates without TA were merged and deformed while the NC-$TA_{0.13}$ maintained their shapes even in vacuum conditions. c) TEM image at high magnification of NC-$TA_{0.13}$ on the red-dotted area in panel (b). d) TEM and HAADF images of NC-$TA_{0.13}$ at the highest magnification. Note: the samples were stained with uranium for the TEM measurement. Sample preparation is described in Supplementary Information 2.15.

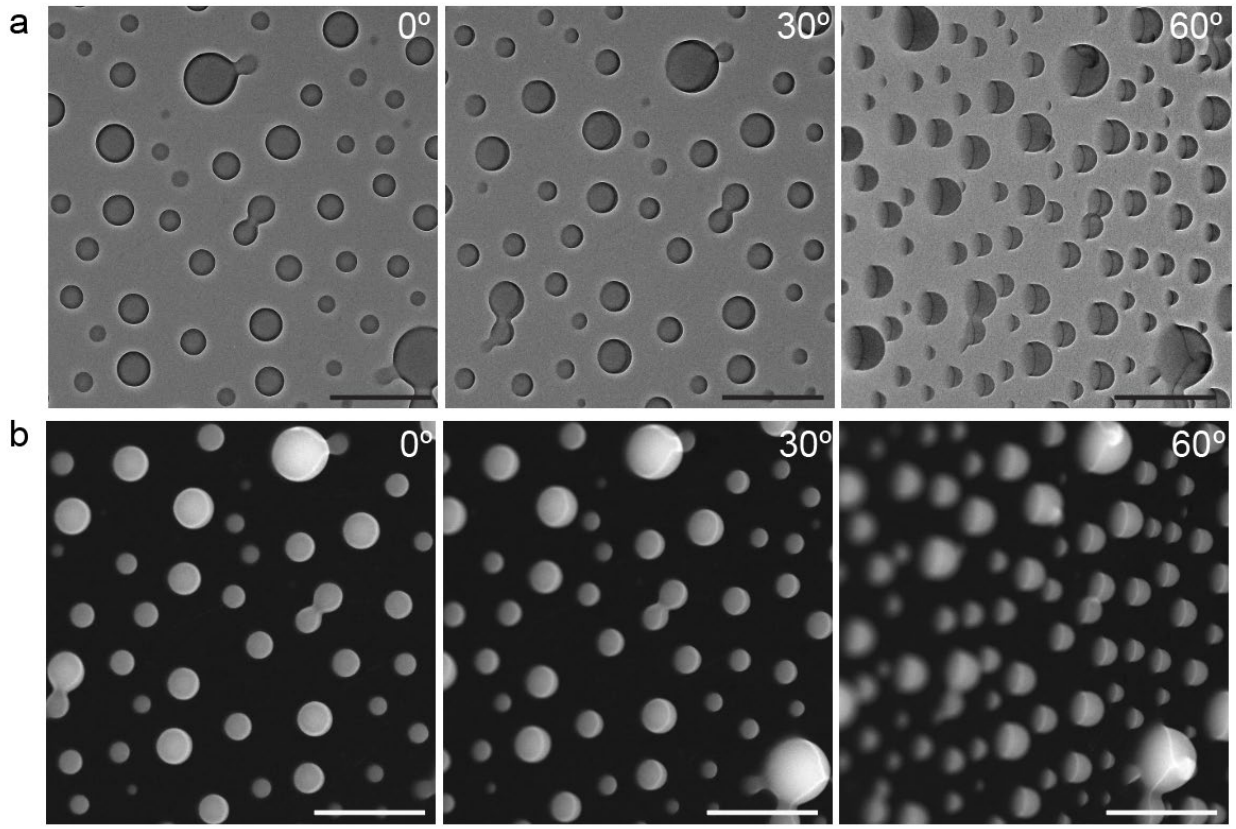

Supplementary Figure 23: TEM and HAADF images at different angles
TEM (a) and HAADF (b) images of NC-$TA_{0.13}$ at different angles of 0° (left) 30° (middle) and 60° (right). The mono-dispersed NC-$TA_{0.13}$ were dried on a TEM grid and imaged at different angles clearly showing the heights of the NC-$TA_{0.13}$. The scale bars indicate 500 nm.

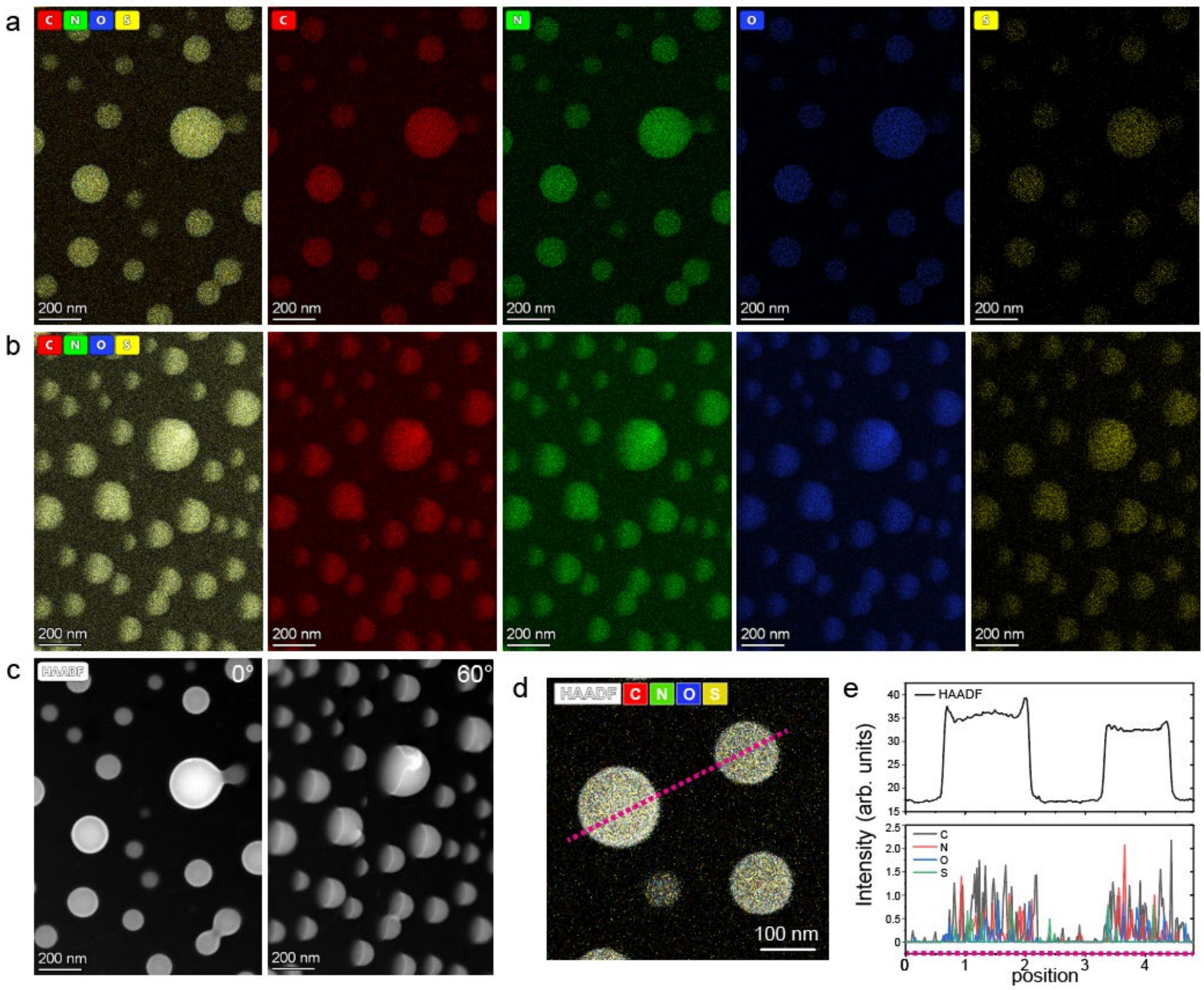

Supplementary Figure 24: EDX mapping of NC-TAs at different angles

EDX mapping of NC-$TA_{0.13}$ at 0° (a) and 60° (b) degrees, showing the highly mono-dispersed NC-$TA_{0.13}$ samples and their heights. c) HAADF image of NC-$TA_{0.13}$ at 0° and 60° degree. d) Merged image of NC-$TA_{0.13}$. e) Signal quantification of HAADF, C, N, O, and S elements shown in a pink-dotted line in (d). The results showed that NC-$TA_{0.13}$ is composed of carbon, nitrogen, oxygen, and sulfur which are major elements of the C5 peptide and heparin.

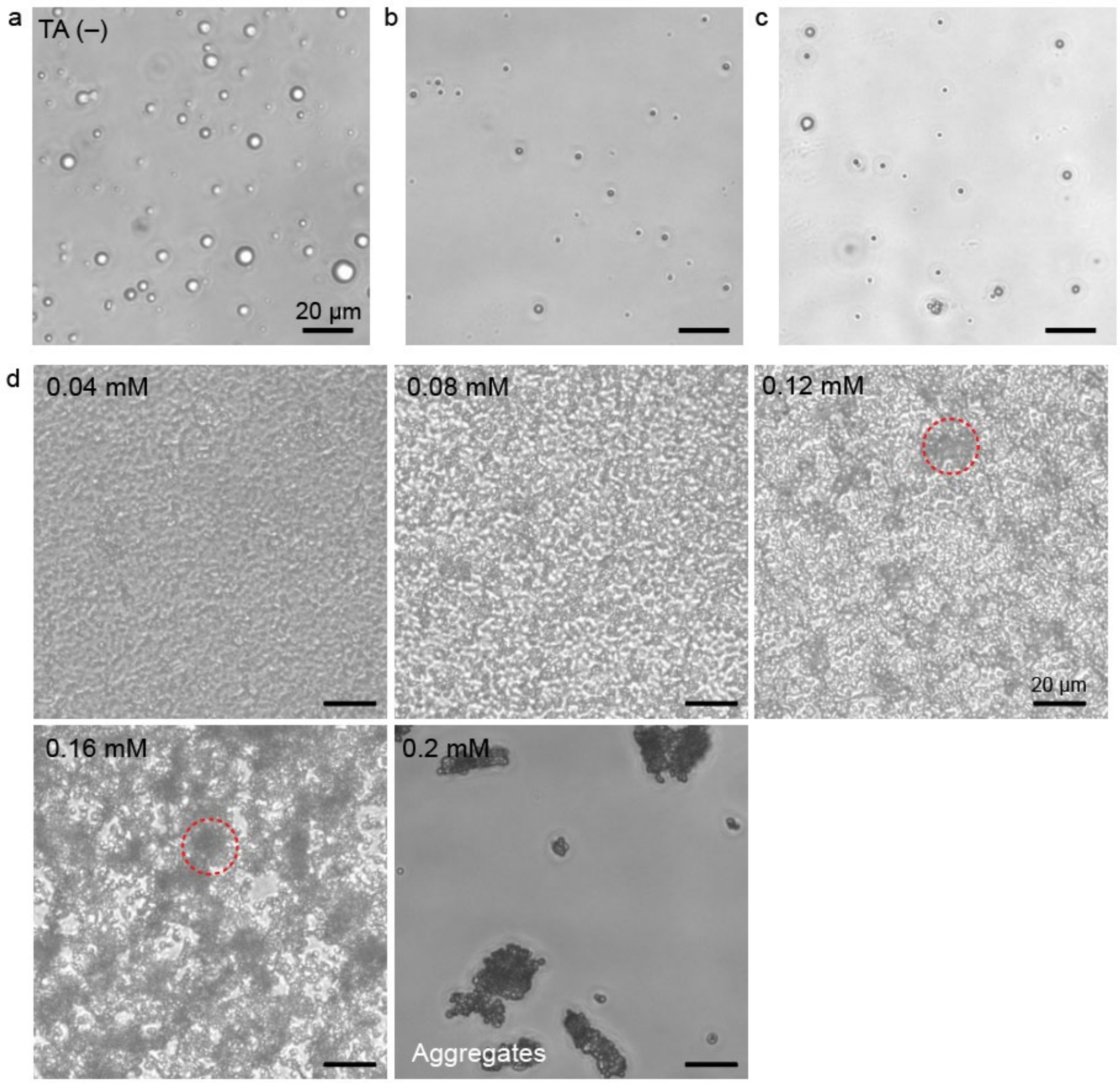

Supplementary Figure 25: Optical images of micro-coacervates after polyphenol encapsulation

Optical images of coacervate droplets before (a) and after TA encapsulation of 0.04 mM (b) and 0.16 mM (c). The images were collected after the centrifugation. After centrifugation, the average diameter of MC-TA was 2.01 ± 0.44 µm. The average and standard deviation represent 16 independent droplets. d) A high concentration of micro-coacervates incubated with different amounts of TA from 0.04 to 0.2 mM overnight. The red-dotted area indicates increased contrast between coacervate droplets due to high TA concentrations. The addition of 0.2 mM TA resulted in the formation of solid aggregates. The images were obtained before centrifugations. The scale bar represents 20 µm.

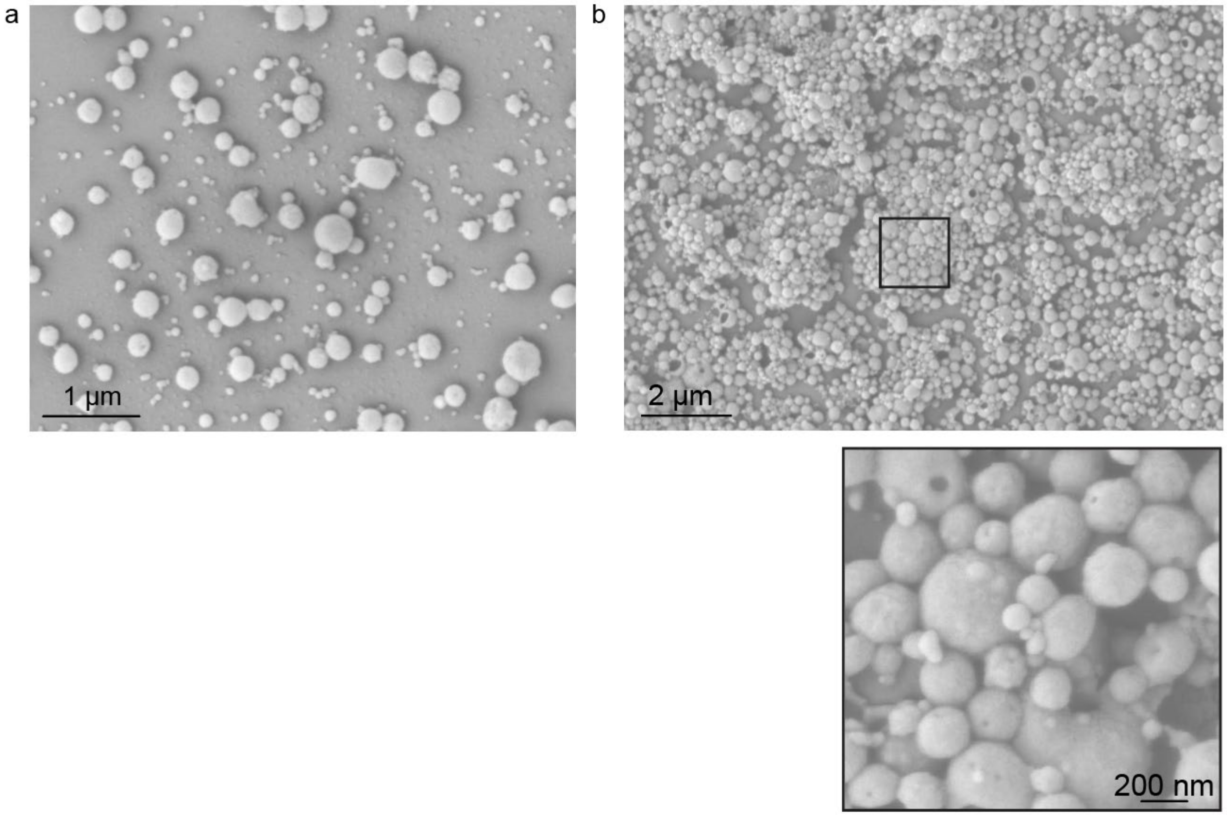

Supplementary Figure 26: SEM images of micro-coacervates

SEM images of low (a) and high (b) concentrations of MC-TAs with different magnifications. TA concentration with 0.16 mM was used for encapsulation. The image in the inset shows the spherical shapes of MC-TAs and size distributions. The average diameters of MC-TAs were 2.01 ± 0.44 μm when hydrated and 0.38 ± 0.9 μm when evaporated, respectively. The average diameter was calibrated using 16 individual MC-TAs from optical and SEM images, respectively. The average and standard deviation represent 16 independent droplets.

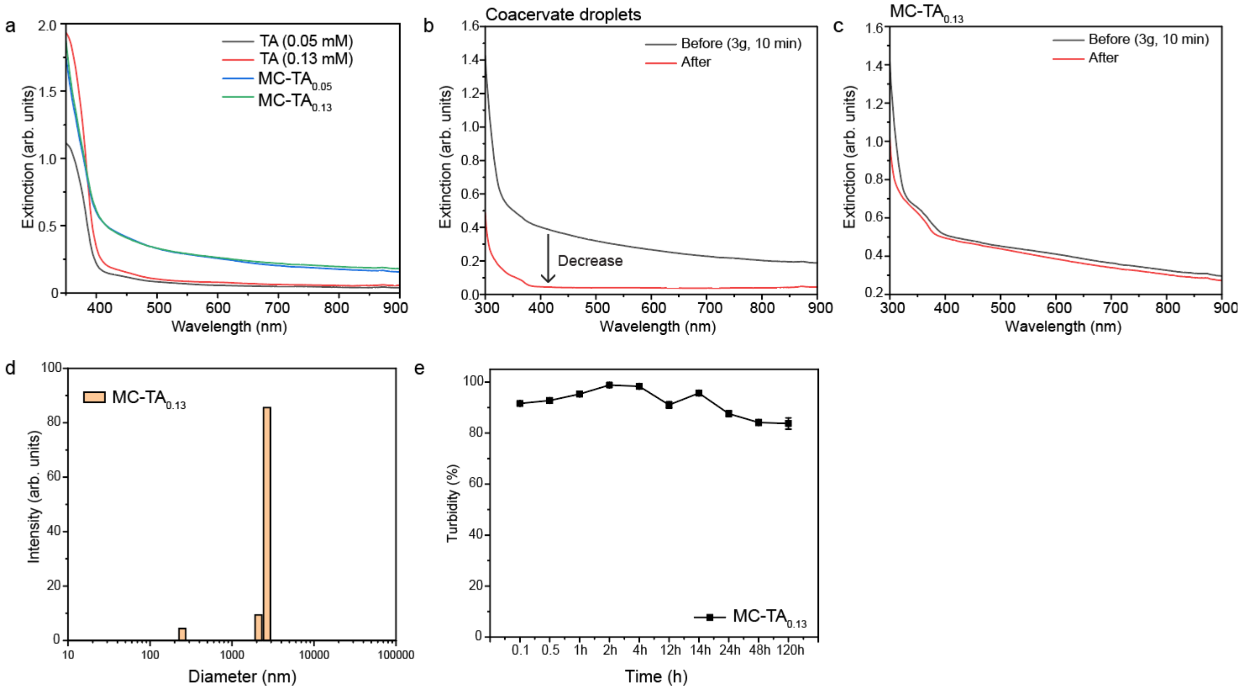

Supplementary Figure 27: TA-encapsulated of micro-coacervates (MC-TAs)

a) UV-vis spectra of MC-TAs and TA only at the concentrations of 0.05 and 0.13 mM. MC-TAs increased extinction ranging from 400 to 900 nm due to the formation of coacervate droplets while TA alone has minimal extinction. Decrease in the extinction of coacervate droplets without TA (b) and MC-$TA_{0.13}$ (c) before and after 3 x g centrifugation for 10 min. The coacervate droplets without TA showed a 98.7% decrease in turbidity while MC-$TA_{0.05}$ decreased by only 1.4%. These results indicate that TA encapsulation improves the stability of not only nano-sized but also micro-sized coacervate droplets. d) DLS data of MC-$TA_{0.13}$. e) Turbidity of MC-$TA_{0.13}$ at different time points, showing its high stability. The turbidity was calibrated based on the extinction value (The equation is described in Supplementary information 2.14).

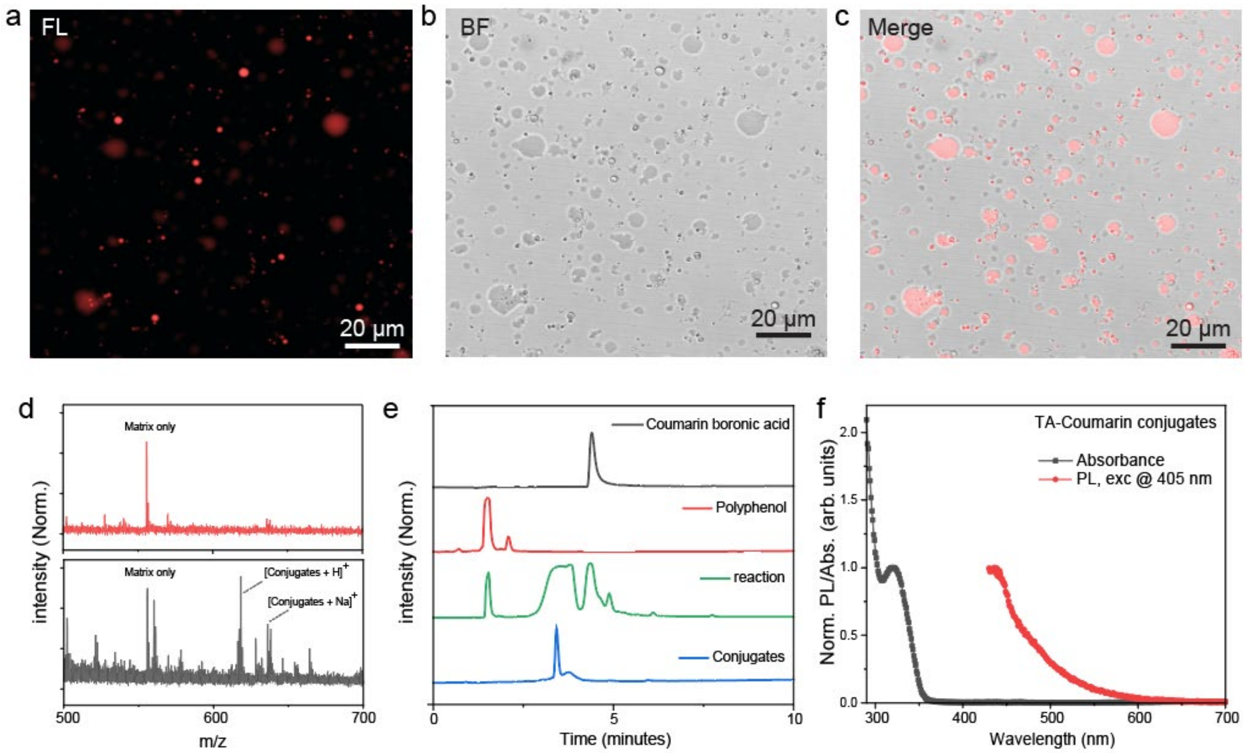

Supplementary Figure 28: Micro-coacervates encapsulating coumarin-TA conjugates
Fluorescence (a), bright field (b), and merge (c) images of MC-TAs which encapsulate TA-coumarin conjugates. MALDI-TOF (d) and HPLC (e) data confirmed polyphenol-coumarin conjugates. Hydroxyl groups in tannic acid can conjugate with coumarin boronic acid, leading to the formation of the boronate ester. After the reaction, the sample was purified using HPLC. f) Normalized absorbance and fluorescence spectrum of TA-coumarin conjugates.

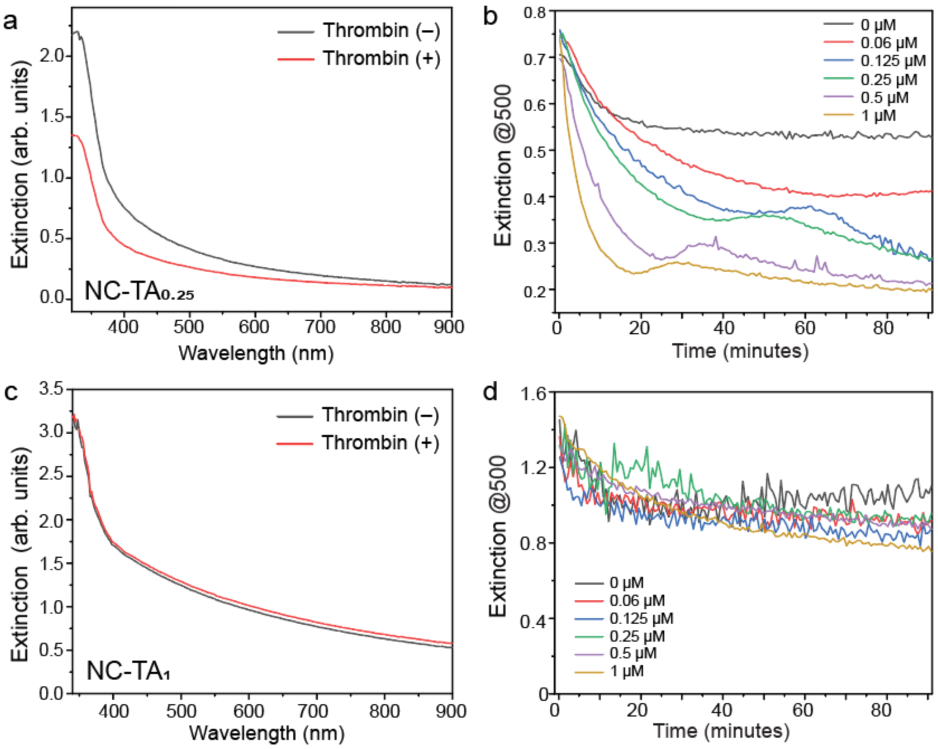

Supplementary Figure 29: UV-vis spectra of NC-TA$_{0.25}$ and NC-TA$_1$

UV-vis spectra and time-dependent changes in the extinction of NC-TA$_{0.25}$ (a-b) and NC-TA$_1$ (c-d). The results indicate that thrombin could disassemble NC-TA$_{0.25,}$ leading to a decrease in extinction while NC-TA$_1$ showed negligible changes in extinction. These results suggest that the increased stability through polyphenol-mediated supramolecular network could lead to a reduction in thrombin proteolytic efficiency. Thrombin with various concentrations ranging from 0.06 to 1 μM was used for the experiment.

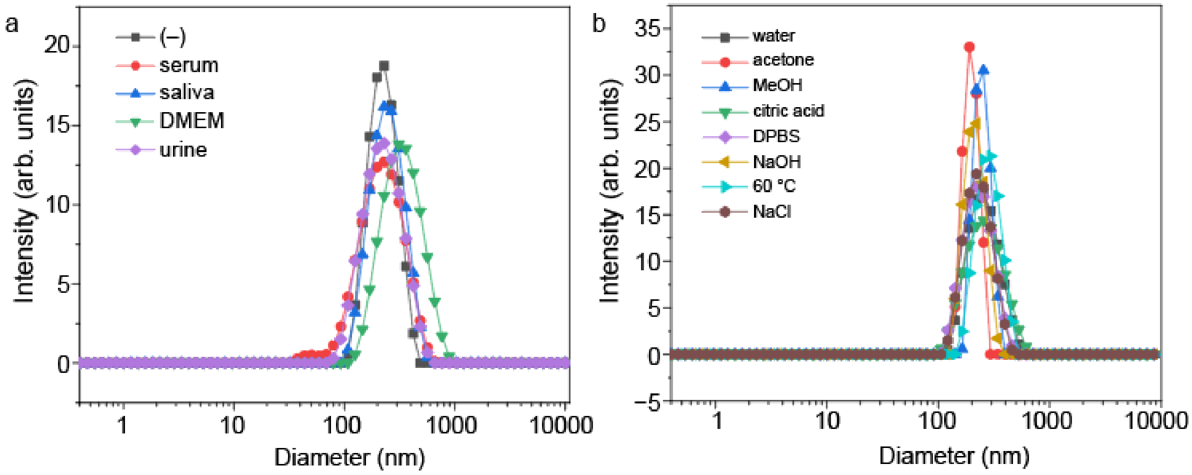

Supplementary Figure 30: Colloidal stability of NC-$TA_{0.13}$ in biofluids and different conditions

Colloidal stability of NC-$TA_{0.13}$ in biofluids (a) and different environments (b). DLS data showed that NC-$TA_{0.13}$ were mono-dispersed (PDI <0.2) and maintained their structural stability in 50% human serum, saliva, urine, and DMEM and different conditions including DPBS, NaOH (pH 10), 60 °C, and NaCl of 150 mM.

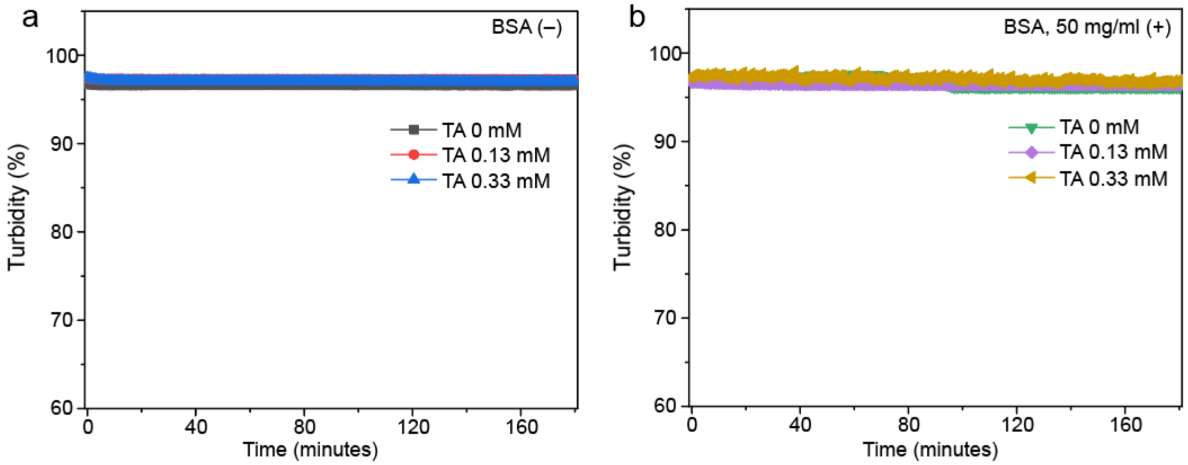

Supplementary Figure 31: Stability test of nano-coacervates in the presence of BSA
Nano-coacervates (*i.e.,* TA: 0 mM), NC-$TA_{0.13}$, and NC-$TA_{0.33}$ were incubated with (a) and without (b) BSA of 50 mg/ml at 37 ℃ for 3 h. The results showed that the coacervates were stable in the presence of the BSA.

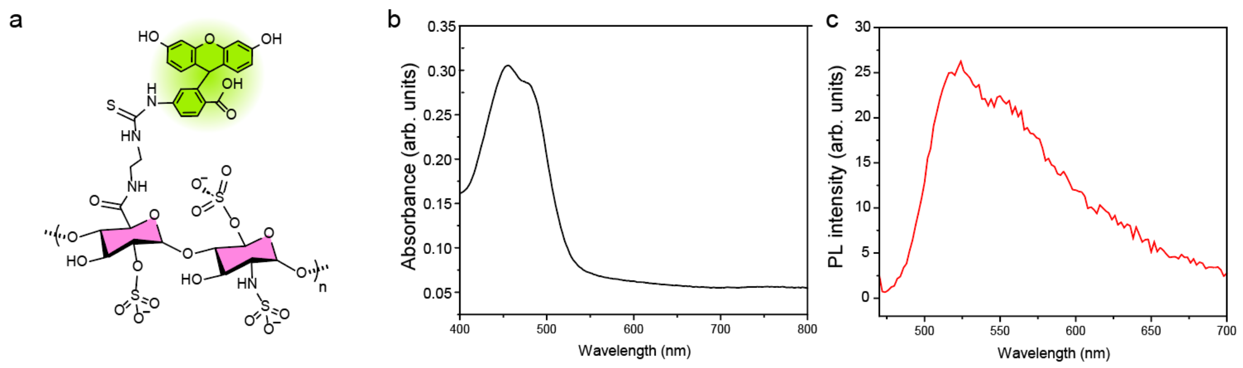

Supplementary Figure 32. Heparin Fluorescein (FITC)
a) Molecular structure of heparin-FITC, Mw 27k (Creative PEGWorks, NC, USA), and its absorbance (b) and fluorescence (c). Heparin-FITC was monitored during the disassembly of nano-coacervates and NC-TAs in human plasma and the addition of thrombin described in Figure 4g.

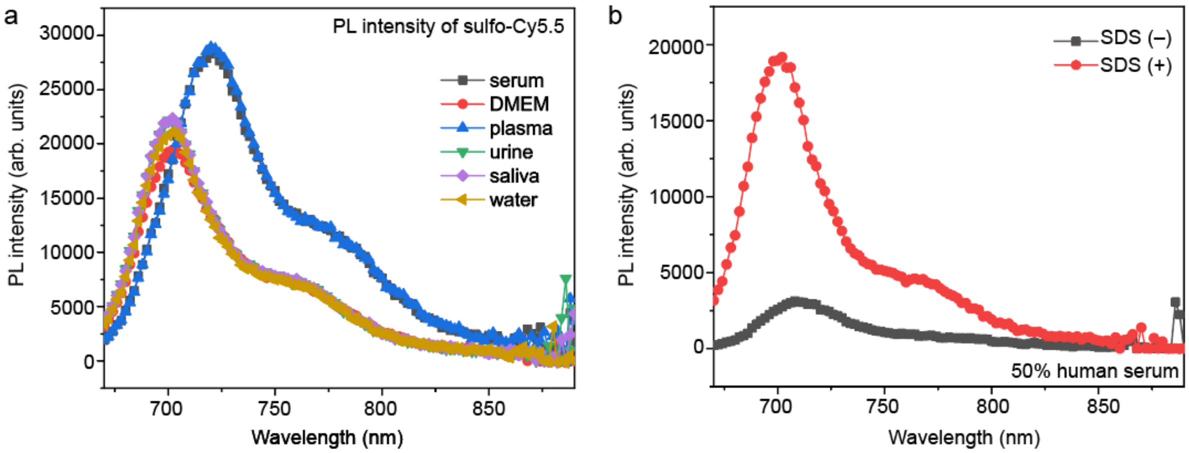

Supplementary Figure 33: No interference of PL intensity of C7 peptide by background medium

a) PL intensity of the C7 (*i.e.,* sulfo-Cy5.5-C5) was evaluated in human serum, plasma, urine, saliva, and DMEM. The results revealed that the biofluids did not quench the fluorescence signal of the sulfo-Cy5.5. b) PL intensity C7-encapsulated NC-$TA_{0.13}$ before and after the addition of SDS (50 mg/ml). PL intensity of the C7 peptide increased as a function of the disassembly of NC-$TA_{0.13}$ induced by the addition of SDS in 50% of human serum.

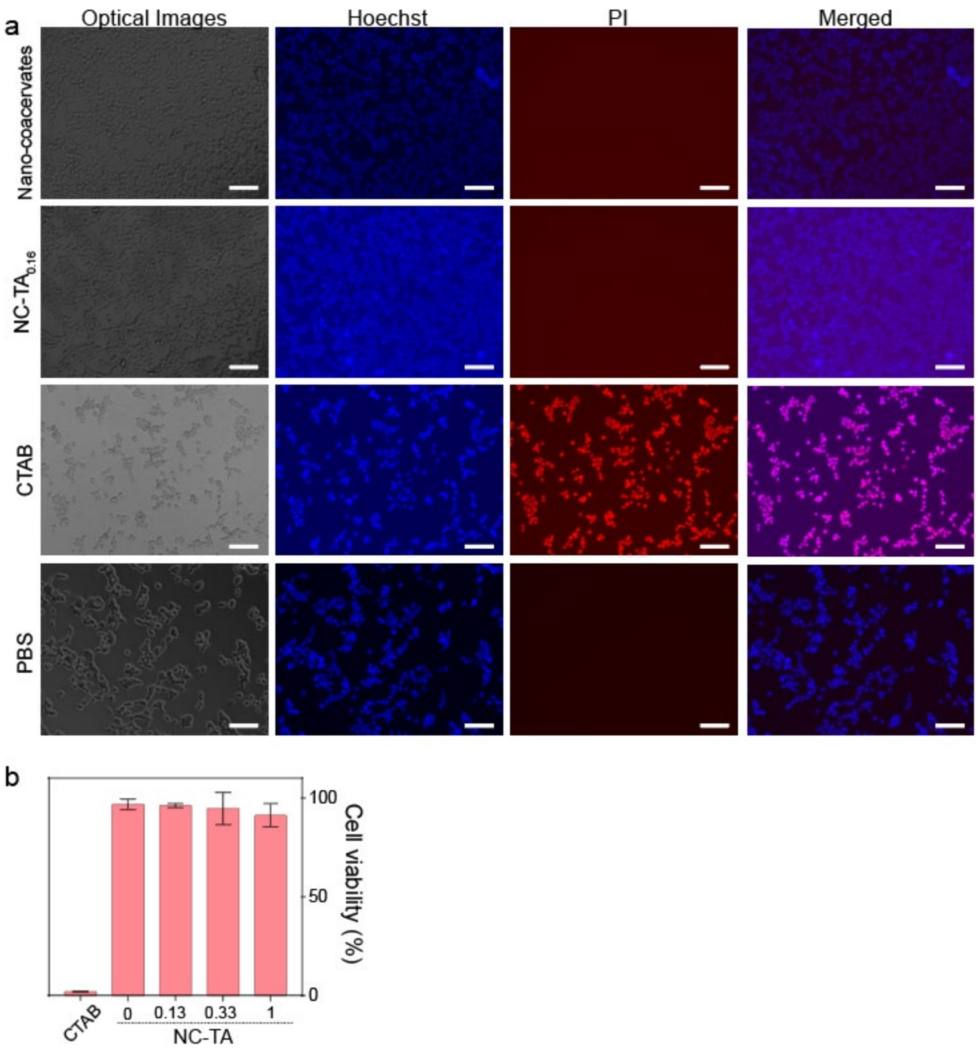

Supplementary Figure 34: Fluorescence images and cell viability of NC, NC-TA$_{0.13}$, CTAB, and PBS

a) Hoechst and PI were used to stain the HEK 293 cells to visualize the cell nucleus and dead cells. CTAB and PBS solution were used as a control (dead *versus* alive cells). Nano-coacervates and NC-TA$_{0.13}$ had negligible fluorescence signal of PI, indicating low cytotoxicity against HEK 293 cells. The scale bars represent 100 µm. b) Cytotoxicity test of NC-TAs, showing high biocompatibility of NC-TAs. Data represent mean ± SD (n = 3).

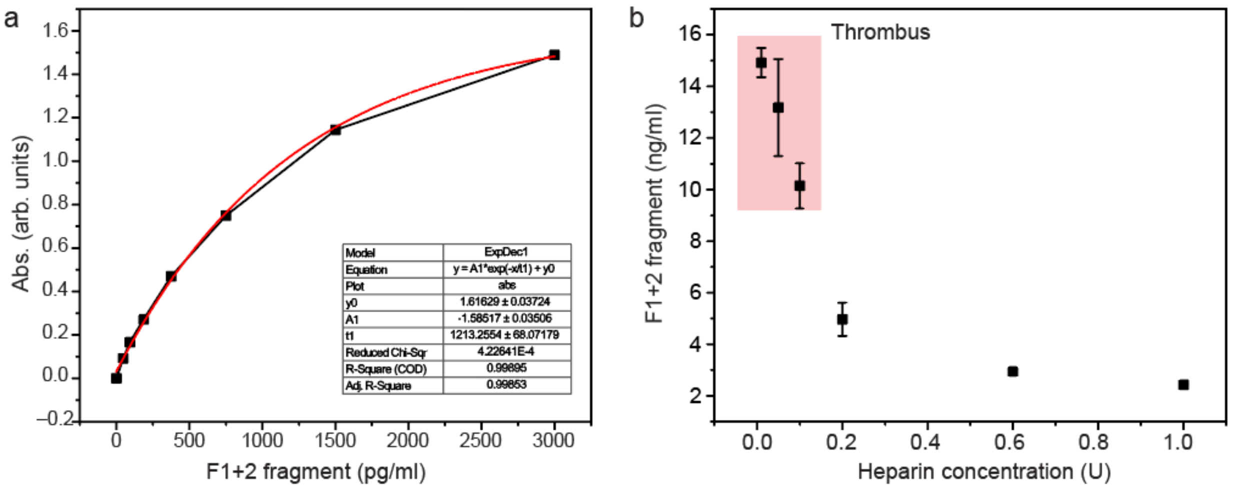

Supplementary Figure 35: Standard curve and F1+2 peptide concentration in human plasma

a) A standard curve of prothrombin fragment F1+2 concentration. Activated optical density (O.D.) at 450 nm was converted to F1+2 concentrations using an equation from the fitting curve. b) F1+2 fragment concentrations from whole human blood at different heparin concentrations from 0.2 to 1 U/ml., showing anticoagulation activity of free heparin. Whole human blood was collected using an EDTA-treated blood collection tube. Calcium chloride was used to trigger blood coagulation. 0.4 mL of whole human blood was incubated with different heparin concentrations. Low heparin concentration (lower than 0.1 U/ml) induced the strong thrombus, increasing F1+2 fragment concentrations due to thrombin activation. Data represent mean ± SD (n = 3).

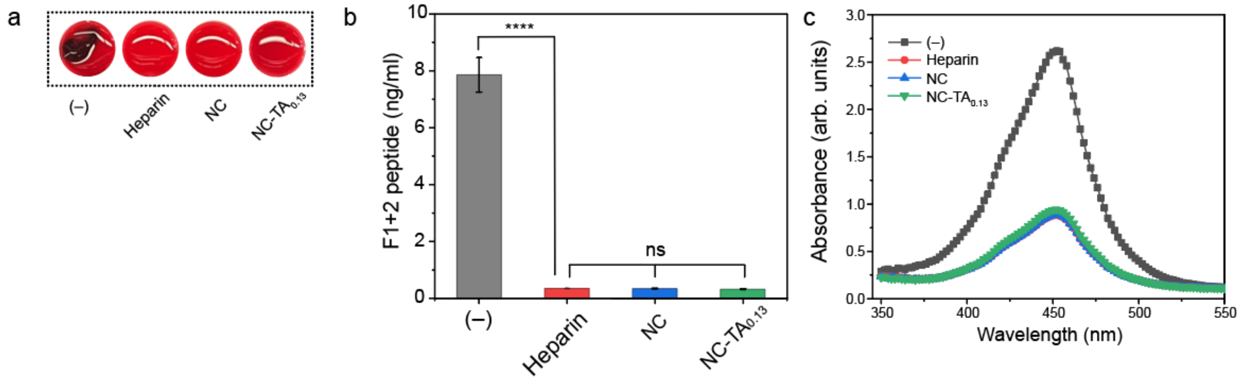

Supplementary Figure 36: ELISA test of NC-TA$_{0.13}$

a) Photographs of whole human blood after incubating with DPBS (–), heparin, nano-coacervates (referred to as NC), and NC-TA$_{0.13}$. The results show that NC and NC-TA$_{0.13}$ prevent blood clots similar to heparin. In contrast, DPBS resulted in blood clotting, thus increasing the concentration of prothrombin fragment F1+2 peptide (expressed in ng/ml) shown in panel (b). Data represent mean ± SD (n = 4). c) Increased absorbance at 450 nm indicates the formation of an antigen-antibody complex of prothrombin fragment F1+2 peptides. The heparin concentration used for this experiment was 40 U/ml. In this study, whole human blood was directly collected with the blood collection tubes that contain heparin, nano-coacervates, and NC-TA$_{0.13}$, respectively. The statistical significance was calculated with the F1+2 peptide of (–), heparin, NC, and NC-TA$_{0.13}$'s *t*-test: **** and ns indicate $p<0.0001$ and non-significance, respectively. Data represent mean ± SD (n = 3).

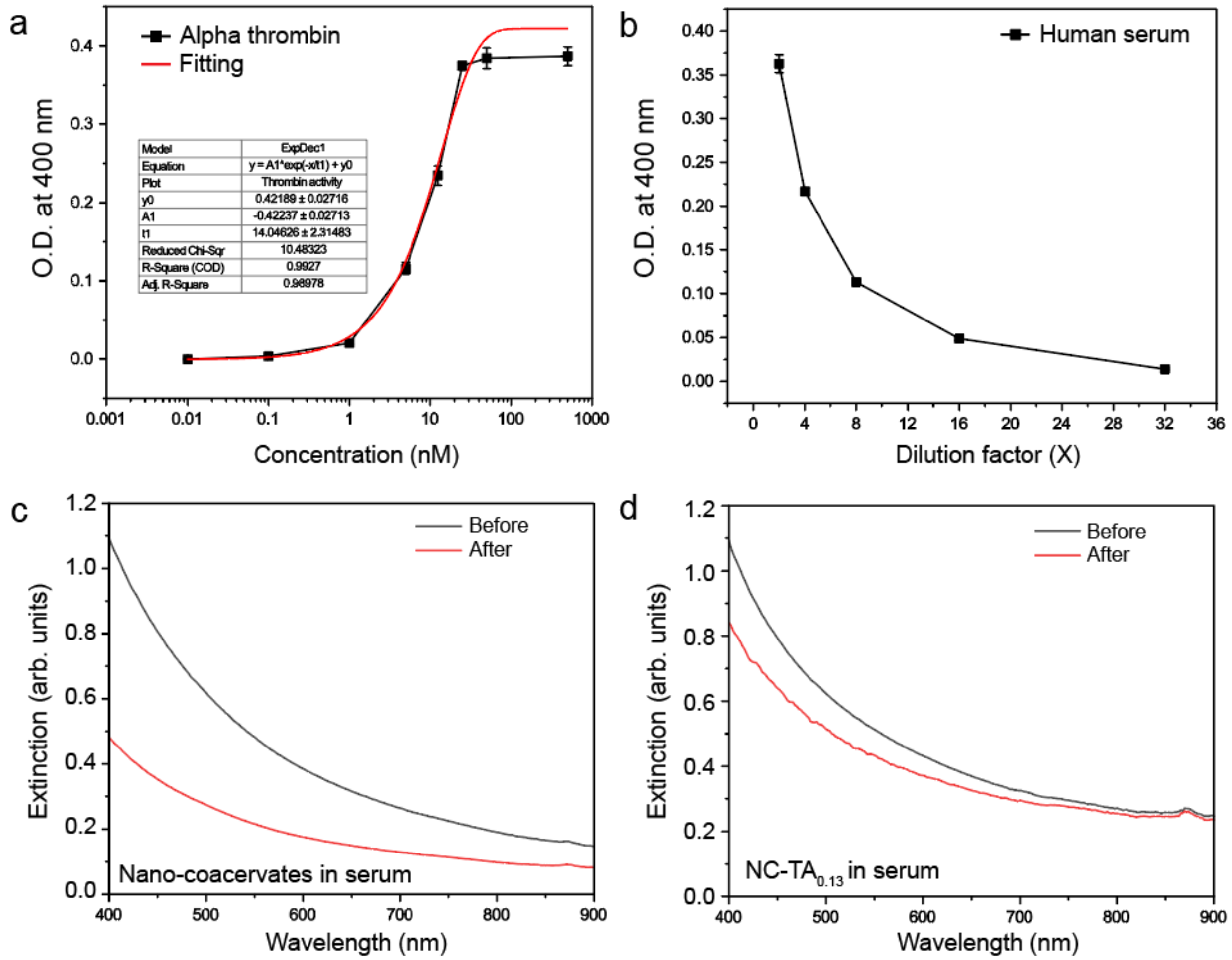

Supplementary Figure 37: Residual thrombin activity and stability test of NC-TA$_{0.13}$ in human serum

a) A standard curve indicates residual thrombin activity of alpha thrombin in different concentrations measured by thrombin chromogenic substrate. b) Optical density (i.e., residual thrombin activity) of human serum samples with serial dilution. Different dilution factors (1:2, 1:4, 1:8, 1:16, and 1:32) were examined, showing that human serum contained residual thrombin activity comparable to that of thrombin at a concentration of 42.5 nM. UV-vis spectra of pristine nano-coacervates (c) and NC-TA$_{0.13}$ (d) in 50% human serum after 1 h incubation. Data in (a) and (b) represent mean ± SD (n = 3).

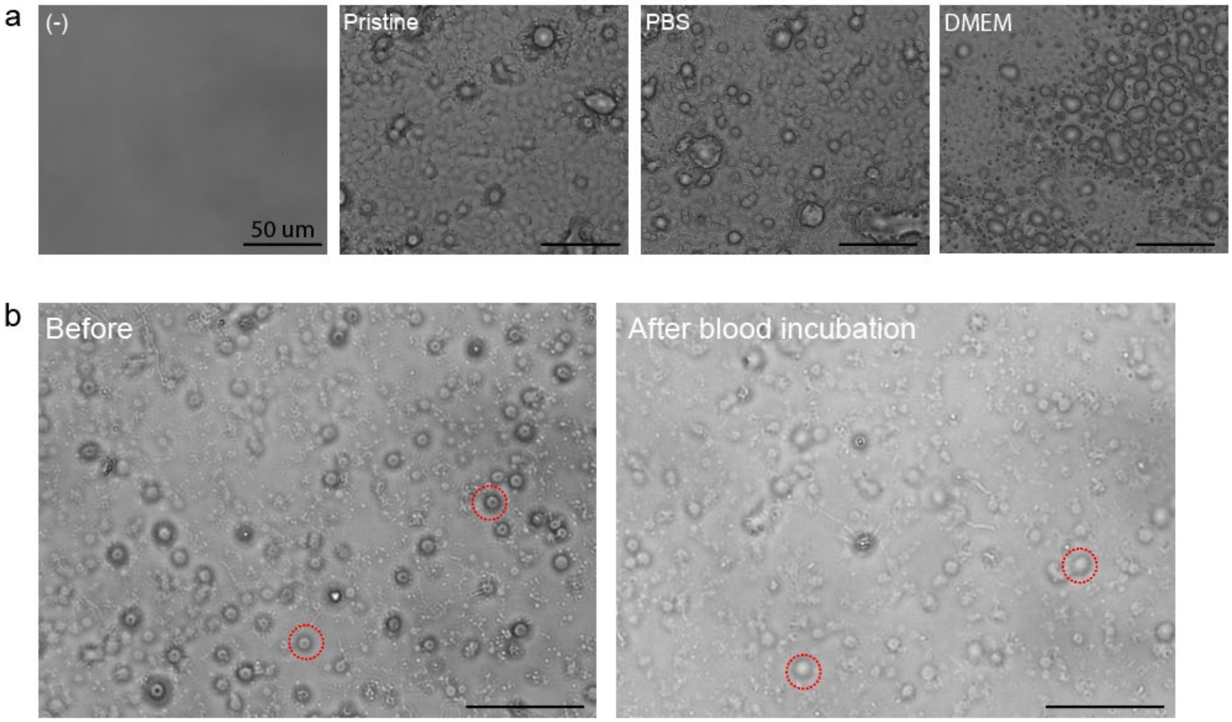

Supplementary Figure 38: Optical images of MC-TAs before and after blood incubation
TA-encapsulated micro-coacervates (MC-TAs) were coated on the glass slides. a) Optical images show the MC-TAs, maintaining spherical shapes on the glass slides after drying and incubating in PBS and DMEM for 1 h. These MC-TAs showed exceptional stability after incubating in whole human blood (100%) (b). The red-dotted circle represents a single MC-TA before and after the incubation. The scale bar represents 50 µm.

5. Supplementary References

1 Jin, Z. *et al.* Endoproteolysis of Oligopeptide-Based Coacervates for Enzymatic Modeling. *ACS nano* (2023).

2 Yim, W. *et al.* Enhanced photoacoustic detection of heparin in whole blood via melanin nanocapsules carrying molecular agents. *ACS nano* **16**, 683-693 (2021).

3 Armbruster, D. A. & Pry, T. Limit of blank, limit of detection and limit of quantitation. *The clinical biochemist reviews* **29**, S49 (2008).

4 Jin, Z. *et al.* Peptide Amphiphile Mediated Co-assembly for Nanoplasmonic Sensing. *Angewandte Chemie* **135**, e202214394 (2023).

5 Johnson, K. A. & Goody, R. S. The original Michaelis constant: translation of the 1913 Michaelis–Menten paper. *Biochemistry* **50**, 8264-8269 (2011).

6 Briggs, G. E. & Haldane, J. B. S. A note on the kinetics of enzyme action. *Biochemical journal* **19**, 338 (1925).

7 Shi, H. *et al.* Fluorescent light-up probe with aggregation-induced emission characteristics for in vivo imaging of cell apoptosis. *Organic & biomolecular chemistry* **11**, 7289-7296 (2013).

8 Neumann, U., Kubota, H., Frei, K., Ganu, V. & Leppert, D. Characterization of Mca-Lys-Pro-Leu-Gly-Leu-Dpa-Ala-Arg-NH2, a fluorogenic substrate with increased specificity constants for collagenases and tumor necrosis factor converting enzyme. *Analytical biochemistry* **328**, 166-173 (2004).

9 Raaijmakers, J. G. Statistical analysis of the Michaelis-Menten equation. *Biometrics*, 793-803 (1987).

10 Borg Andersson, A., Birkhed, D., Berntorp, K., Lindgärde, F. & Matsson, L. Glucose concentration in. *Eur J Oral Sci* **106**, 931-937 (1998).

11 Guthrie Jr, R. D. & Hines Jr, C. Use of Intravenous Albumin in the Critically Ill Patient. *American Journal of Gastroenterology (Springer Nature)* **86** (1991).

12 Schlimp, C. J. *et al.* Rapid measurement of fibrinogen concentration in whole blood using a steel ball coagulometer. *Journal of Trauma and Acute Care Surgery* **78**, 830-836 (2015).

13 Ota, S. *et al.* Elevated levels of prothrombin fragment 1+ 2 indicate high risk of thrombosis. *Clinical and Applied Thrombosis/Hemostasis* **14**, 279-285 (2008).

14 McElfresh, C., Harrington, T. & Vecchio, K. S. Application of a Novel New Multispectral Nanoparticle Tracking Technique. *Meas. Sci. Technol.* **29**, 065002 (2018).

15 Di Cera, E. Thrombin. *Molecular aspects of medicine* **29**, 203-254 (2008).

16 Lee, H., Dellatore, S. M., Miller, W. M. & Messersmith, P. B. Mussel-inspired surface chemistry for multifunctional coatings. *science* **318**, 426-430 (2007).

17 Wang, J. *et al.* A nanoscale tool for photoacoustic-based measurements of clotting time and therapeutic drug monitoring of heparin. *Nano letters* **16**, 6265-6271 (2016).
