# Supplementary material for: Polyphenol-stabilized coacervates for enzyme-triggered drug delivery
Source: Nat Commun. 2024 Aug 24;15:7295. doi: 10.1038/s41467-024-51218-8 (PMC11344779; doi:10.1038/s41467-024-51218-8)
Supplement: Supplementary file 3 — Description of Additional Supplementary Files [file 41467_2024_51218_MOESM3_ESM.pdf]

Description of Additional Supplementary Files

File Name: Supplementary Movie 1
Description: C1 peptide with heparin, showing no coacervate formation.

File Name: Supplementary Movie 2
Description: C2 peptide with heparin, forming nano-coacervates.

File Name: Supplementary Movie 3
Description: C3 peptide with heparin, forming micro-coacervates.
